# Auxin Response Factor SlARF2 Is an Essential Component of the Regulatory Mechanism Controlling Fruit Ripening in Tomato

**DOI:** 10.1371/journal.pgen.1005649

**Published:** 2015-12-30

**Authors:** Yanwei Hao, Guojian Hu, Dario Breitel, Mingchun Liu, Isabelle Mila, Pierre Frasse, Yongyao Fu, Asaph Aharoni, Mondher Bouzayen, Mohamed Zouine

**Affiliations:** 1 University of Toulouse, INPT, Laboratory of Genomics and Biotechnology of Fruit, Castanet-Tolosan, France; 2 INRA, UMR990 Génomique et Biotechnologie des Fruits, Chemin de Borde Rouge, Castanet-Tolosan, France; 3 Weizmann Institute of Science, Department of Plant Sciences, Faculty of Biochemistry, Rehovot, Israel; The University of North Carolina at Chapel Hill, UNITED STATES

## Abstract

Ethylene is the main regulator of climacteric fruit ripening, by contrast the putative role of other phytohormones in this process remains poorly understood. The present study brings auxin signaling components into the mechanism regulating tomato fruit ripening through the functional characterization of *Auxin Response Factor2* (*SlARF2*) which encodes a downstream component of auxin signaling. Two paralogs, *SlARF2A* and *SlARF2B*, are found in the tomato genome, both displaying a marked ripening-associated expression but distinct responsiveness to ethylene and auxin. Down-regulation of either *SlARF2A* or *SlARF2B* resulted in ripening defects while simultaneous silencing of both genes led to severe ripening inhibition suggesting a functional redundancy among the two ARFs. Tomato fruits under-expressing *SlARF2* produced less climacteric ethylene and exhibited a dramatic down-regulation of the key ripening regulators *RIN*, *CNR*, *NOR* and *TAGL1*. Ethylene treatment failed to reverse the non-ripening phenotype and the expression of ethylene signaling and biosynthesis genes was strongly altered in *SlARF2* down-regulated fruits. Although both SlARF proteins are transcriptional repressors the data indicate they work as positive regulators of tomato fruit ripening. Altogether, the study defines SlARF2 as a new component of the regulatory network controlling the ripening process in tomato.

## Author Summary

The plant hormone ethylene is regarded as the major regulator of fruit ripening but the putative role of other hormones remains elusive. Auxin Response Factors (ARFs) are transcriptional regulators modulating the expression of auxin-response genes shown recently to play a primary role in regulating fruit set in tomato, but the potential role of ARFs in the ripening process is still unknown. We show that among all tomato ARF genes, SlARF2 displays the most remarkable ripening-associated pattern of expression, which prompted its functional characterization. Two paralogs, SlARF2A and SlARF2B are identified in the tomato that are shown to be functionally redundant. The simultaneous down-regulation of SlARF2A/B genes leads to a severe ripening inhibition with a dramatically reduced ethylene production and a strong decrease in the expression of key regulators of fruit ripening such as *rin* and *nor*. The study defines SlARF2 as a new component of the regulatory network controlling the ripening process in tomato, suggesting that auxin, in concert with ethylene, might be an essential hormone for fruit ripening. While providing a new insight into the mechanisms underlying the control of fleshy fruit ripening, the study uncovers new avenues towards manipulating the ripening process through means that have not been described so far.

## Introduction

Fruit ripening is a complex, genetically programmed process that is associated with dramatic metabolic and textural transformations including color change, fruit softening, sugar accumulation and production of flavor and aroma compounds [1–3]. The ripening process ultimately leads to fruit withering allowing dispersal of the seeds and based on their ripening mechanism, fleshy fruits are divided into climacteric and non-climacteric types [4]. Climacteric fruit ripening is characterized by the autocatalytic increase in ethylene biosynthesis, and it is widely accepted that this hormone acts as main trigger and coordinator of the ripening process [5]. In support of this view, several genes involved in ethylene metabolism and signaling have been shown to be essential for fruit ripening in tomato and reducing ethylene production via suppression of ethylene biosynthesis genes, ACC synthase (*ACS*) and ACC oxidase (*ACO*), leads to the inhibition of fruit ripening [6–9]. Likewise, the tomato *Never-ripe* (*Nr*) mutant, bearing an altered allele of the ethylene receptor gene *ETR3*, also shows a non-ripening phenotype due to its reduced ethylene sensitivity [10,11]. In line with the ETR receptors being negative regulators of ethylene signaling, silencing of either *LeETR4* or *LeETR6* with a fruit-specific promoter causes enhanced ethylene sensitivity and early ripening phenotype [12]. On the other hand, repression of tomato EIN3-Binding Factors SlEBF1/SlEBF2, the downstream component of ethylene signaling F-BOX proteins responsible for the degradation of EIN3 protein, causes constitutive ethylene responses and early fruit ripening [13]. In concert with ethylene, the control of fruit ripening relies on other key regulators, some of which have been functionally characterized. In this regard, silencing of the homeobox protein LeHB1 results in delayed ripening [14] and *MADS-box* genes like *RIPENING-INHIBITOR* (*RIN*) and *TOMATO AGAMOUS-LIKE 1* (*TAGL1*) are proved to dramatically affect fruit ripening [15–18]. The *COLORLESS NON-RIPENING* (*CNR*), a SQUA-MOSA promoter binding protein (SBP), is shown to directly influence the expression of *RIN* and other *MADS-box* genes during fruit ripening [19,20]. Moreover, fruits in the *rin* and *cnr* mutants remain firm and green for an extended period, and they are deficient in ethylene production and unable to ripen upon exogenous ethylene treatment [19,21]. Besides its important role in fruit ripening, ethylene is also involved in several other plant developmental processes [22].

Without minimizing the role of ethylene, it has long been considered that other plant hormones are likely to play a critical role for both the attainment of competence to ripen and the coordination of subsequent steps of the ripening process. In this regard, assumptions that fruit ripening is most likely driven by a complex hormonal balance have been formulated for a long time in the literature, even though clear experimental evidence supporting this hypothesis remained lacking. Auxin is among the first to be assigned a role in the ripening of fleshy fruits because auxin treatment of mature fruit was shown to delay ripening [23–27]. More direct evidence for the involvement of auxin in ripening came recently through the implementation of reverse genetic strategies targeting auxin-dependent transcriptional regulators [28–32]. Auxin signaling is known to regulate the expression of target genes mainly through two types of transcriptional regulators, namely, Aux/IAAs and Auxin Response Factors (ARF). While Aux/IAAs are known to be repressors of auxin-dependent gene transcription, ARFs can be either transcriptional activators or repressors via direct binding to the promoter of auxin-responsive genes [33–39]. In the tomato, 22 ARFs have been identified [39] and the accumulation of some ARF transcripts has been reported to be under ethylene regulation during tomato fruit development suggesting that auxin signaling may influence the control of climacteric fruit ripening [28]. Recently, it was shown that SlARF4 plays a role in fruit ripening mainly by controlling sugar metabolism, and down-regulation of this ARF resulted in ripening-associated phenotypes such as enhanced firmness and chlorophyll content leading to dark green fruit and blotchy ripening [28,32,40].

The marked ripening-associated pattern of expression of *SlARF2* prompted the investigation of its physiological significance and in particular its putative role in fleshy fruit development and ripening. Since two putative co-orhtologs of Arabidopsis ARF2 have been identified in the tomato, named *SlARF2A* and *SlARF2B*, transgenic lines were generated that are specifically silenced either in one or simultaneously in the two *ARF2* paralogs (S2 Fig). *SlARF2* down-regulated lines displayed strong ripening defects and the expression of key regulators of fruit ripening, such as *RIN*, *CNR*, *NOR* and *TAGL1* was markedly decreased in *SlARF2* under-expressing lines which position ARF2 as a new component of the regulatory network controlling the ripening process in tomato.

## Results

### Sl-ARF2 is encoded by two genes with distinct expression patterns in the tomato

**Fig 1:**
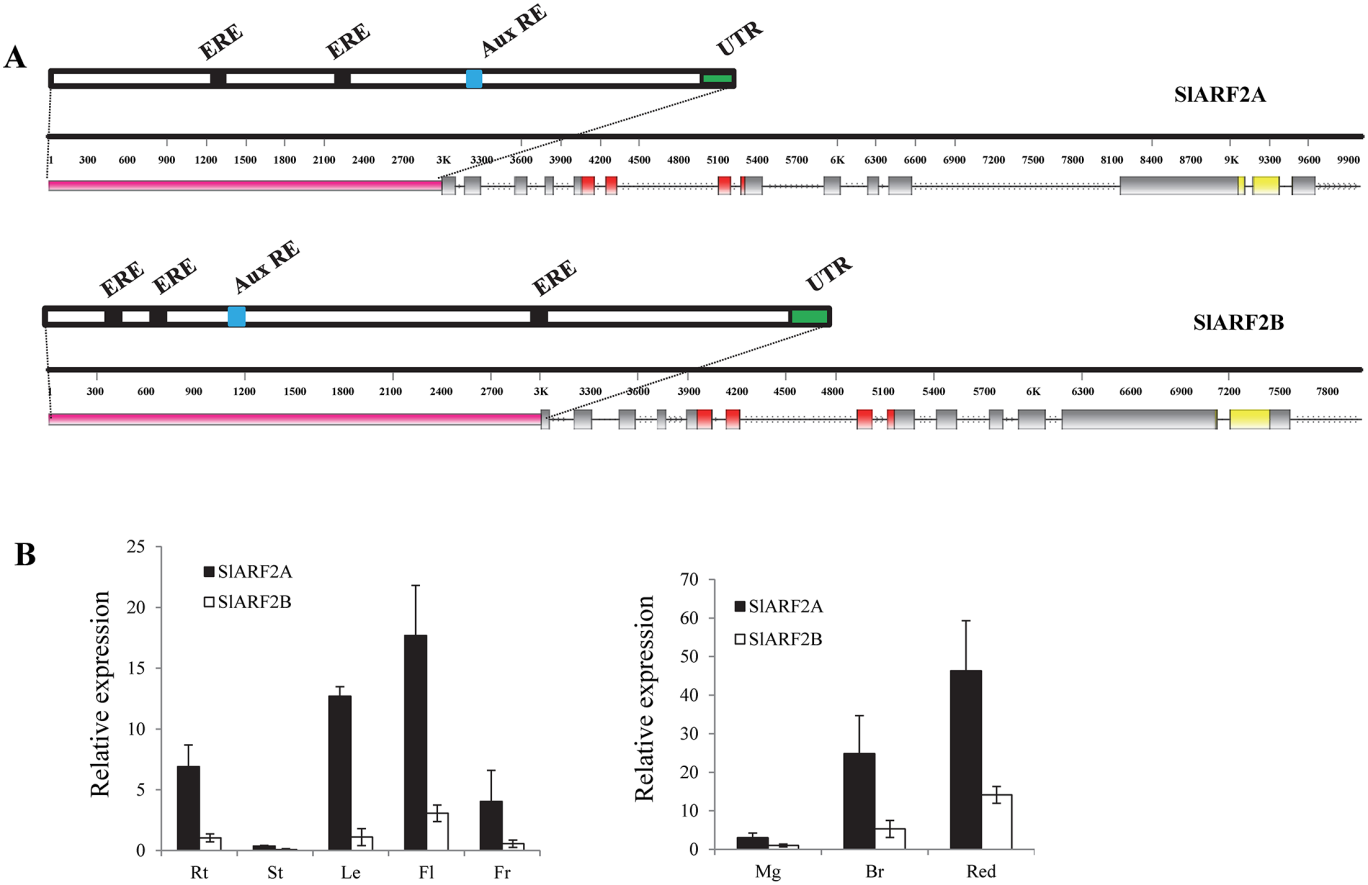
Structural features and expression patterns of tomato *SlARF2A* and *SlARF2B* genes. (A) Genomic structure analysis of *SlARF2A* and *SlARF2B* genes were drawn using Fancy gene V1.4 software (http://bio.ieo.eu/fancygene/) and *SlARF2A SlARF2B* iTAG2.40 gene model data. The pink portion represents the promoter region; the strandlines represent intron parts; the gray boxes indicate exon parts; the yellow boxes region responsible for dimerization with Aux/IAA proteins (domain III and IV); the red boxes correspond to the DNA binding domain (DBD); ERE and AuxRE correspond to the ethylene and auxin responsive *cis*-elements. (B) Expression pattern of *SlARF2A/2B* monitored by quantitative real-time RT-PCR (qPCR) in total RNA samples extracted from root (Rt), stem (St), leaf (Le), flower (Fl), fruit (Fr), mature green fruit (MG), breaker fruit (Br) and red fruit (Re). Relative mRNA levels corresponding to *SlARF2A*/*SlARF2B* genes were normalized against actin in each RNA sample. The relative mRNA levels of *SlARF2B* in root and at mature green (MG) stage were used as reference (relative mRNA level 1). Error bars mean ±SD of three biological replicates.

Some members of the *ARF* gene family were shown to play a role in regulating important aspects of tomato fruit ripening [28,32]. More recently, expression profiling of tomato ARFs revealed that some members of this gene family display a ripening-associated increase of transcript accumulation suggesting their potential involvement in regulating this process [39]. Among these, the expression pattern of ARF2 is appealing which prompted its molecular and functional characterization. In contrast to Arabidopsis where a single ARF2 gene is present, two putative orthologs are found in the tomato genome with *SlARF2A* (Solyc03g118290.2.1) being located in chromosome 3 and *SlARF2B* (Solyc12g042070.1.1) in chromosome 12 [39]. The two genomic clones share similar structural organization with, however, *SlARF2A* being made of 15 exons while only 14 exons are present in *SlARF2B*. The isolation of full-length cDNAs corresponding to *SlARF2A* (2541 bp) and *SlARF2B* (2490 bp) indicated that the deduced protein sizes are 847 and 830 amino acids, respectively (Table 1), and pairwise comparison of the two SlARF2 protein sequences revealed 83.3% amino acid identity. The search for protein domains in Expasy database (http://prosite.expasy.org/) indicated the presence of highly conserved domains typical of ARFs (Fig 1A) including the DBD (DNA Binding Domain) and the dimerization domains (protein/protein domain III and IV). Moreover, the analysis of a 2 kb promoter sequence using PLACE/signal search tool (http://www.dna.affrc.go.jp/PLACE/signalscan.html) revealed the presence of putative Ethylene Response (ERE) and Auxin Response (AuxRE) elements in both *SlARF2A* and *SlARF2B* promoters (Fig 1A).

**Table 1:** Main structural features of the tomato *SlARF2A* and *SlARF2B*.

*Nomenclature*	*Gene*			*Predicted Protein*		*Domains*	
*SlARF2*	*iTAG Gene ID*	*Exons*	*Introns*	*Length*	*MW (kDa)*	*DBD*	*Dimerization domain*
*SlARF2A*	Solyc03g118290.2.1	15	14	847 aa	94.01358	146–248	721–803
*SlARF2B*	Solyc12g042070.1.1	14	13	830 aa	92.46828	128–230	704–785

Assessing transcript accumulation by quantitative-RT-PCR confirmed the ripening-associated patterns of expression of the two *SlARF2* genes (Fig 1B). *SlARF2A* and *SlARF2B* are expressed in all plant tissues tested including root, leaf, stem, flower and fruit with, however, a notably higher transcript accumulation for *SlARF2A* in both vegetative and reproductive tissues. It is noteworthy that the transcript levels corresponding to the two *ARF2* genes undergo a net up-regulation at the onset of fruit ripening (Fig 1B) suggesting that SlARF2A and SlARF2B may play an active role in this developmental process.

### *SlARF2A* and *SlARF2B* are differentially regulated by auxin and ethylene

**Fig 2:**
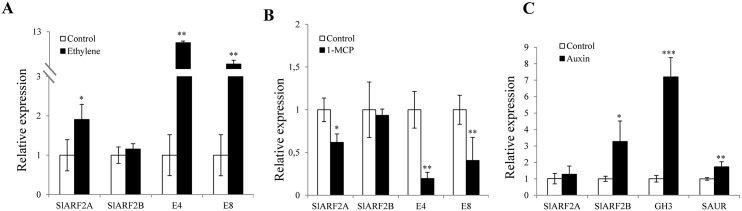
Auxin and ethylene responsiveness of *SlARF2A* and *SlARF2B* genes. (A) qPCR analysis of *SlARF2A* and *SlARF2B* transcripts in total RNA samples extracted from wild-type mature green fruits treated with 50 ml.L^-1^ ethylene for 5 hours. (B) qPCR analysis of *SlARF2A* and *SlARF2B* transcripts in total RNA samples extracted from wild-type breaker fruits treated with 1-MCP (1.0 mg.L^-1^) for 16 hours. (C) qPCR analysis of *SlARF2A* and *SlARF2B* transcripts in total RNA samples extracted from wild-type mature green fruits treated with 20 μM IAA or buffer (control) for 6 hours. The relative mRNA levels of *SlARF2A*/*SlARF2B* genes were normalized against actin. The results were expressed using control untreated fruit as reference with relative mRNA level set to 1. Error bars mean ±SD of three biological replicates. Stars indicate the statistical significance using Student’s t-test: *0.01 < p-value < 0.05, ** 0.001< p-value <0.01, *** p-value < 0.001. *E4*, *E8*: ethylene response genes; *GH3*, *SAUR*: auxin response genes.

The presence of conserved AuxRE and ERE *cis*-regulatory elements in the promoter region of *SlARF2A* and *SlARF2B* and the expression of both genes in developmental processes known to be regulated by both auxin and ethylene prompted the investigation of their responsiveness to the two hormones. Transcript accumulation assessed by RT-qPCR indicated that *SlARF2A*, but not *SlARF2B*, is responsive to exogenous ethylene treatment in mature green fruit (Fig 2A), and that this ethylene-induced expression is repressed by 1-MCP, the inhibitor of ethylene perception (Fig 2B). By contrast, *SlARF2B* expression was up-regulated by auxin in mature green fruit, while that of *SlARF2A* showed no responsiveness to auxin treatment (Fig 2C). Genes known to be ethylene (*E4*, *E8*) or auxin (*GH3*, *SAUR*) responsive were used as controls to validate the efficacy of the hormone treatment.

### SlARF2A and SlARF2B are nuclear localized and act as transcriptional repressors of auxin-responsive genes

**Fig 3:**
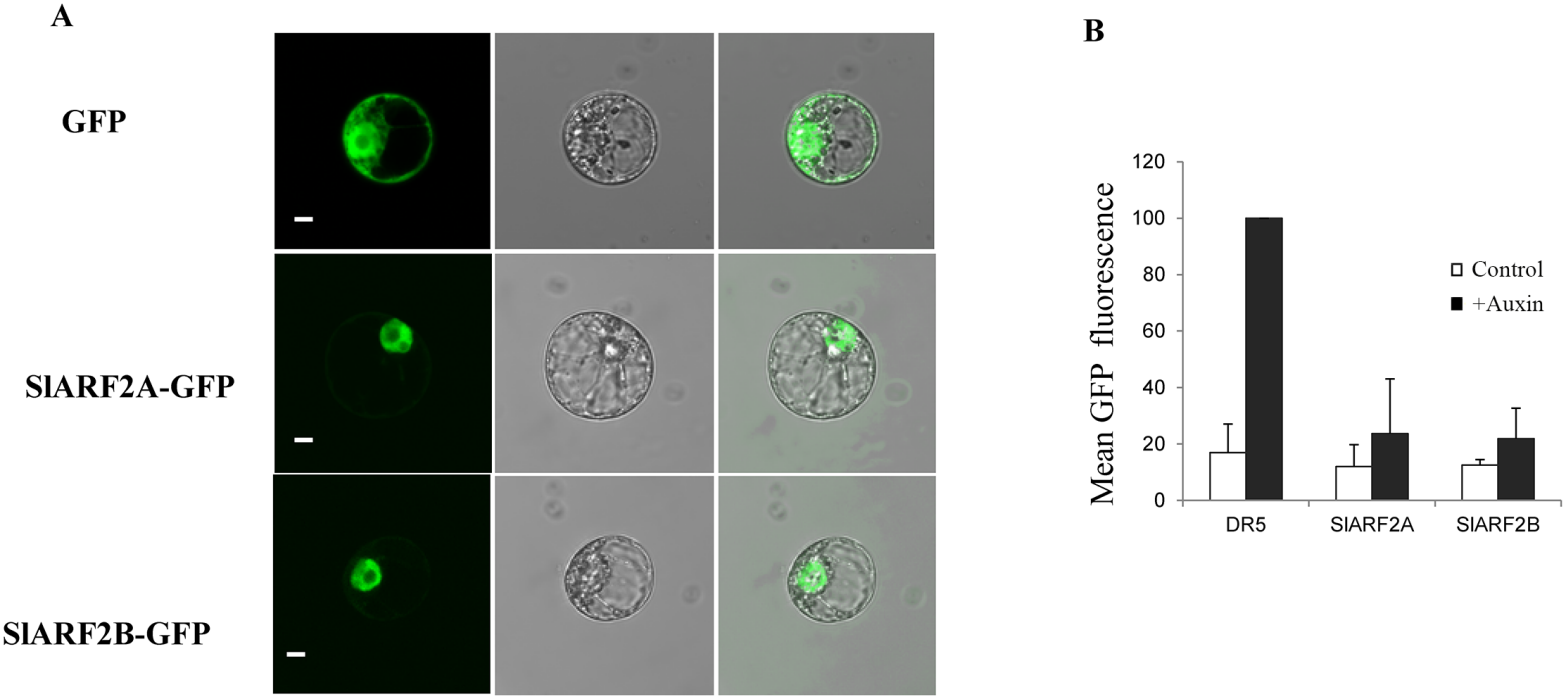
Subcellular localization and functional analysis of SlARF2A and SlARF2B by single cell system. (A) Subcellular localization of tomato SlARF2A/2B proteins. SlARF2A/2B-GFP fusion proteins were transiently expressed in BY-2 tobacco protoplasts and subcellular localization was analyzed by confocal laser scanning microscopy. The merged pictures of the green fluorescence channel (left panels) and the corresponding bright field (middle panels) are shown in the right panels. The scale bar indicates 10 μm. The top pictures correspond to control cells expressing GFP alone. The middle and bottom pictures correspond to cells expressing the SlARF2A-GFP and SlARF2B-GFP fusion proteins, respectively. (B) SlARF2A/2B protein represses the activity of DR5 *in vivo*. SlARF2A/2B proteins were challenged with a synthetic auxin-responsive promoter called *DR5* fused to the GFP reporter gene. A transient expression assay using a single cell system was performed to measure the reporter gene activity. Tobacco protoplasts were transformed either with the reporter construct (DR5::GFP) alone or with both the reporter and effector constructs (35S::SlARF2A/2B) and incubated in the presence or absence of 50 μM 2,4-D. GFP fluorescence was measured 16 h after transfection. For each assay, three biological replicates were performed. GFP mean fluorescence is indicated in arbitrary unit (a.u.) ± standard error.

The subcellular localization of SlARF2A and SlARF2B proteins was then assessed using translational fusion to the Green Fluorescent Protein (GFP) in a tobacco protoplast transient expression assay. Microscopy analysis clearly showed that SlARF2A/2B:GFP fusion proteins exclusively localized into the nucleus (Fig 3A), consistent with their putative role in transcriptional regulation activity. The ability of SlARF2A/2B proteins to regulate the activity of auxin-responsive promoters was then evaluated in a single cell system. A reporter construct, consisting of the synthetic auxin-responsive promoter DR5 fused to GFP [41], was co-transfected into tobacco protoplasts with an effector construct allowing the constitutive expression of SlARF2A or SlARF2B protein. As expected the DR5-driven GFP expression was strongly enhanced by auxin (2,4-D) treatment. However, the presence of either SlARF2A or SlARF2B proteins strongly inhibited this auxin-induced activity of DR5 promoter, clearly demonstrating that SlARF2A and SlARF2B act *in vivo* as strong transcriptional repressors of auxin-dependent gene transcription (Fig 3B).

### Generation of *SlARF2A-RNAi*, *SlARF2B-RNAi*, and *SlARF2AB-RNAi* lines in tomato

**Fig 4:**
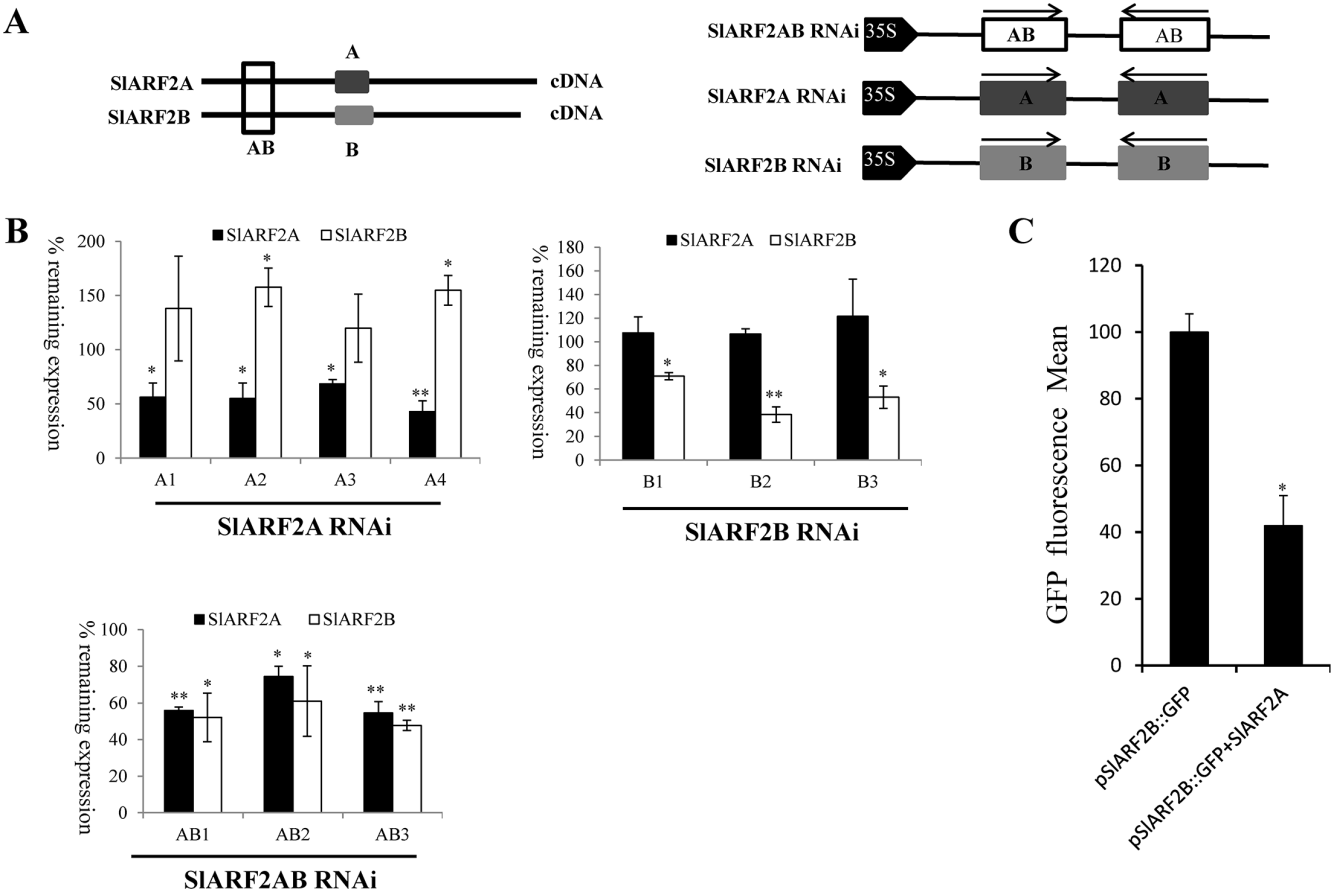
Expression pattern of *SlARF2A* and *SlARF2B* in *SlARF2 RNAi* transgenic lines. (A) *SlARF2A-RNAi*, *SlARF2B-RNAi* and *SlARF2AB-RNAi* constructs. AB = specific fragment in the DBD binding domain for both SlARF2A and SlARF2B used in *SlARF2AB-RNAi* construct. A = specific fragment in the middle region of SlARF2A used in *SlARF2A-RNAi* construct, B = specific fragment in the middle region of SlARF2B used in *SlARF2B-RNAi* construct. (B) transcript levels of *SlARF2A* and *SlARF2B* in *RNAi* transgenic lines analyzed by quantitative RT-PCR. Expression of *SlARF2A*/*SlARF2B* in wild type was taken as reference (relative mRNA level 100%) and the *SlActin* gene as an internal control. % remaining expression of *SlARF2A* and *SlARF2B* transcript levels relative to the reference. Error bars mean ±SD of three biological replicates. Stars indicate a statistical significance (p<0.05) using Student’s t-test. (C) SlARF2A negatively regulates the activity of *SlARF2B* promoter. Tobacco protoplasts were transformed either with the reporter construct (pSlARF2B::GFP) alone or with both the reporter and effector constructs (35S-SlARF2A) and GFP fluorescence was measured 16 h after transfection. Effector construct lacking SlARF2A was used as control for the co-transfection experiments. Transformations were performed in triplicate. Mean fluorescence is indicated in arbitrary unit (a.u.) ± standard error. Stars indicate a statistical significance (Student’s t-test): * p-value < 0.05, ** p-value < 0.01.

To gain insight into the physiological significance of SlARF2, transgenic lines under-expressing the two paralogs were generated in the MicroTom tomato genetic background. To this purpose, dedicated RNAi constructs were designed to selectively target either *SlARF2A* or *SlARF2B* allowing the generation of transgenic lines specifically silenced in only one of the two *SlARF2* genes (Fig 4A). Transgenic RNAi lines in which both paralogs are simultaneously silenced were also obtained. Repression of *SlARF2A* and *SlARF2B* in the RNAi lines was confirmed by qPCR analyses in seedlings and fruit tissues showing that the accumulation of *SlARF2A* or *SlARF2B* transcripts was selectively reduced in the appropriate silenced lines whereas in the *SlARF2A*/*2B* double knockdown lines both *SlARF2* genes were significantly down-regulated (Fig 4B). Importantly, the expression of the most closely related ARFs in terms of sequence identity was not reduced in *SlARF2A*/*2B* transgenic lines, thus ruling out a lack of specificity of the RNAi strategy (S2 Fig).

It is noteworthy that, in the *SlARF2A-RNAi* lines the down-regulation of *SlARF2A* seems to be compensated by an increase in *SlARF2B* expression, while such a compensation mechanism does not occur in the *SlARF2B-RNAi* lines. To check whether SlARF2A may be directly involved in the transcriptional regulation of *SlARF2B*, a GFP reporter construct driven by the *SlARF2B* promoter was co-transfected into tobacco protoplasts with an effector construct allowing constitutive expression of SlARF2A. The data clearly show that the presence of SlARF2A inhibits the expression of the *GFP* reporter gene driven by the *SlARF2B* promoter, revealing the ability of SlARF2A to repress *in vivo* the transcriptional activity of *SlARF2B* (Fig 4C).

### Down-regulation of *SlARF2* results in enhanced expression of auxin-responsive genes

**Fig 5:**
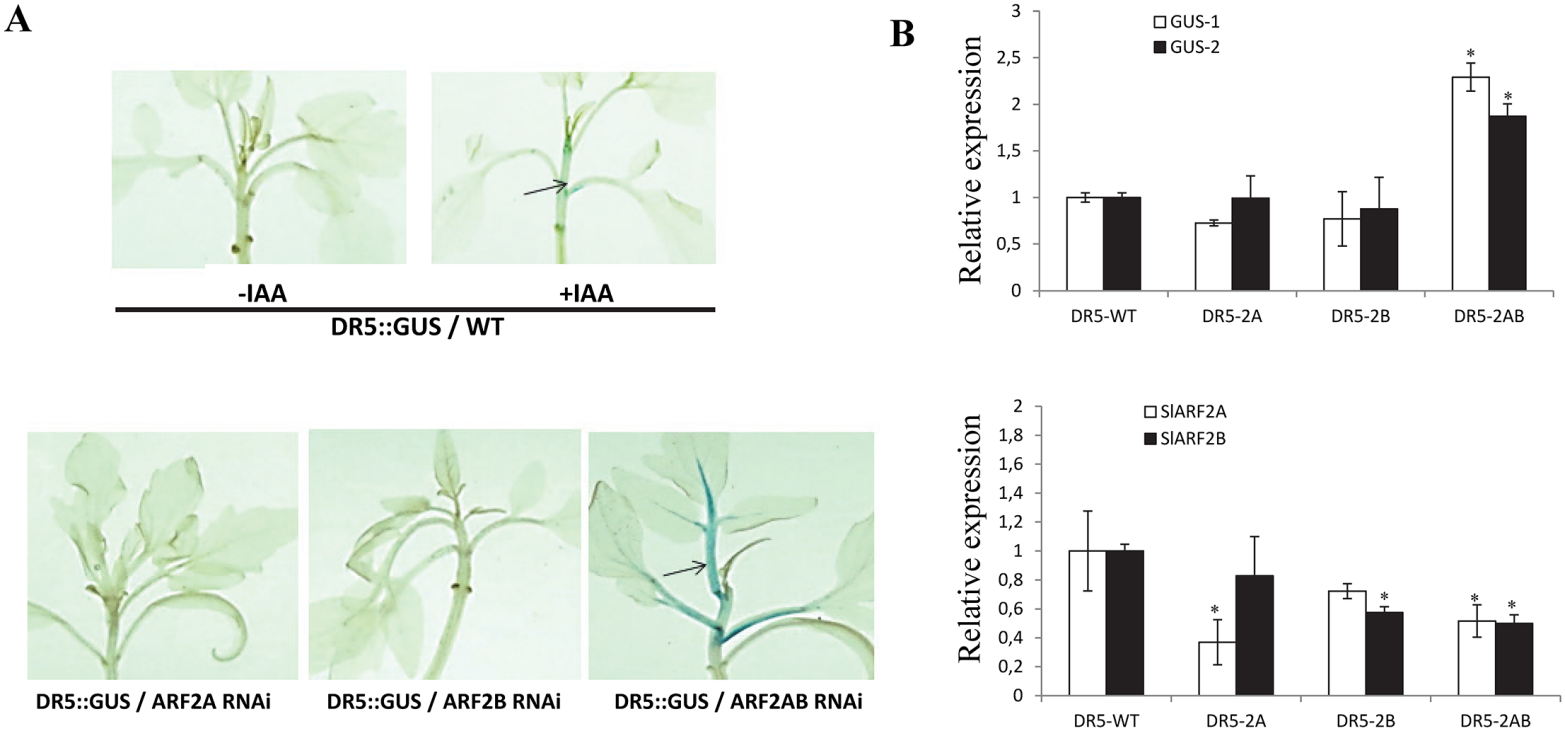
Impact of the down-regulation of *SlARF2A* and *SlARF2B* on auxin response assessed *in planta* following genetic crosses between DR5::GUS and *SlARF2* down-regulated lines. (A) Expression pattern of the GUS reporter gene under the control driven by the auxin-inducible DR5 promoter in wild type (WT) and *SlARF2* down-regulated genetic background. Seedlings were treated with auxin (IAA 20 μM for 3 hours) or with a mock solution. Upper panel: *in planta* expression of the GUS reporter gene driven by DR5 in WT genetic background in the absence (left) or presence (right) of auxin treatment. Bottom panel: Expression of the GUS reporter gene driven by DR5 in *ARF2A RNAi* (left), *ARF2B RNAi* (middle) and *ARF2AB RNAi* (right) genetic background. (B) Quantitative RT-PCR expression analysis of *GUS* and *SlARF2A/2B* genes in WT and *SlARF2A* and *SlARF2B-RNAi* lines crossed with DR5::GUS lines. The relative mRNA levels of *GUS-1/GUS-2* (Upper panel) and *SlARF2A/2B* (bottom panel) in wild type were standardized to 1.0, referring to the *SlActin* gene as internal control. Error bars mean ±SD of three biological replicates. *0.01 < p-value < 0.05. DR5-WT = DR5::GUS/WT; DR5-2A = DR5::GUS/ARF2A RNAi; DR5-2B = DR5::GUS/ARF2B RNAi; DR5-2AB = DR5::GUS/ARF2AB RNAi. *GUS-1* and *GUS-2* refer to the use of two distinct pairs of primers designed in two distinct regions of the GUS mRNA sequence.

*SlARF2A/B* down-regulated lines displayed multiple auxin-related phenotypes including triple cotyledon formation and enhanced root branching (S1 Fig) supporting the idea that the reduced expression of ARF2 might affect auxin responses. To investigate whether SlARF2A and SlARF2B are involved in auxin responses *in planta*, genetic crosses were performed between the *SlARF2 RNAi* lines and a tomato line expressing the GUS reporter gene under the control of the DR5 auxin-responsive promoter. In the wild-type background, the basal expression of the DR5-driven GUS was low but displayed a net increase upon exogenous auxin treatment (Fig 5A). By contrast, the basal expression of the GUS reporter gene was dramatically high in the *SlARF2AB-RNAi* background in the absence of auxin treatment indicating that the under-expression of SlARF2 results in enhanced expression of the auxin-responsive gene. Interestingly, such an increase in GUS expression was not observed neither in *SlARF2A-RNAi* nor in *SlARF2B-RNAi* background, suggesting that the two genes are functionally redundant and can compensate for each other (Fig 5A). Assessing GUS transcript accumulation by qPCR confirmed the higher expression of the DR5-driven GUS in the *SlARF2AB-RNAi* background but not in the *SlARF2A* and *SlARF2B-RNAi* lines (Fig 5B).

### *SlARF2 RNAi* fruits display altered ripening phenotypes

**Fig 6:**
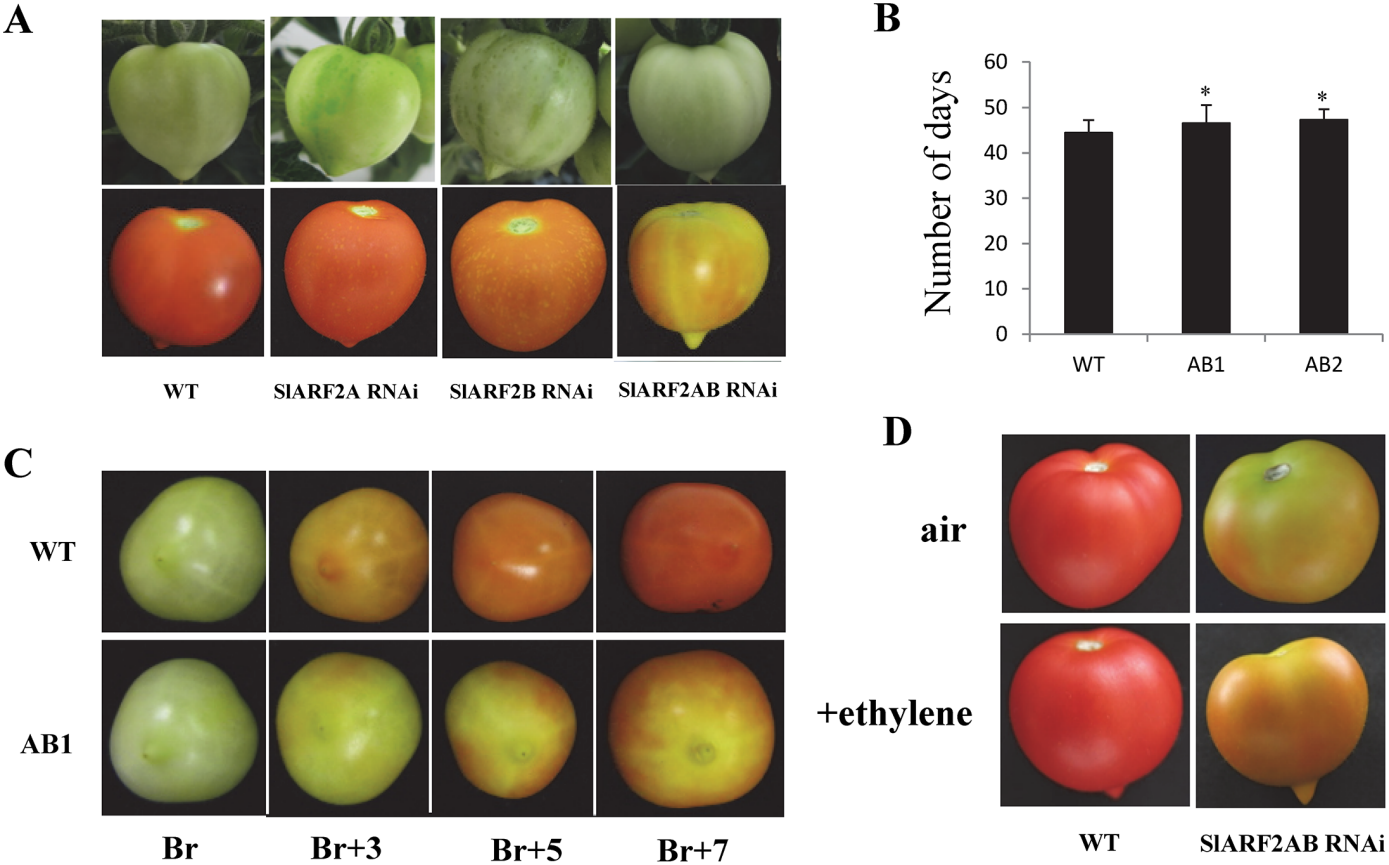
Altered ripening phenotypes of *SlARF2* down-regulated lines. (A) Ripening phenotypes of *SlARF2A-RNAi; SlARF2B-RNAi* and *SlARF2AB-RNAi* fruits at mature green (upper panel) and ripe (lower panel) stages. The *SlARF2A/SlARF2B-RNAi* fruits show spiky phenotype at mature green stage and ripe stage fruits, *SlARF2AB-RNAi* mutant displays inhibited ripening. (B) Time (number of days) from anthesis to breaker in wild type and two independent *SlARF2AB-RNAi* lines. (C) Ripening phenotypes of wild-type (WT) and *SlARF2AB-RNAi* fruits. Transgenic fruits never reach a full red color. Br = breaker stage; Br+3 = 3 days post-breaker stage; Br+5 = 5 days post-breaker stage; Br+7 = 7 days post-breaker stage. (D) Effect of ethylene treatment on wild type (WT) and *SlARF2AB-RNAi* fruit. Mature green fruits from WT and *SlARF2AB-RNAi* lines were treated 2 hours and 3 times per day with 10 ppm ethylene or with air for 3 days. After 7 days, both ethylene treated and untreated wild type fruit reached full red while *SlARF2AB-RNAi* fruits treated or untreated displayed orange sectors on the fruit surface and never get red.

Considering the ripening-associated pattern of both *SlARF2A* and *SlARF2B*, we sought to analyze the fruit phenotypes of *SlARF2A* and *SlARF2B* single and double knockdown tomato lines. In both *SlARF2A* and *SlARF2B-RNAi* single knockdown lines, the fruit exhibited dark green spots at immature and mature green stages, and then displayed a mottled pattern of ripening with yellow/orange spots on the skin remaining till the full mature stage (Fig 6). The double silenced lines exhibited more severe ripening defects with yellow and orange patches never reaching the typical red color of wild type or out-segregating lines, again suggesting that SlARF2A and SlARF2B may have redundant function in fruit ripening (Fig 6A). Assessing the time period from anthesis to breaker stage revealed a slight but statistically significant delay (2 to 3 days delay) in the onset of ripening between wild type and double knockdown lines (Fig 6B). The fruit color in *SlARF2AB-RNAi* lines never get fully red (Fig 6C) and full ripening cannot be recovered upon exogenous ethylene treatment of the *SlARF2A/B RNAi* double knockdown fruits which suggests a possible alteration in ethylene perception or response (Fig 6D).

### SlARF2A and SlARF2B affect ethylene production and perception in the fruit

**Fig 7:**
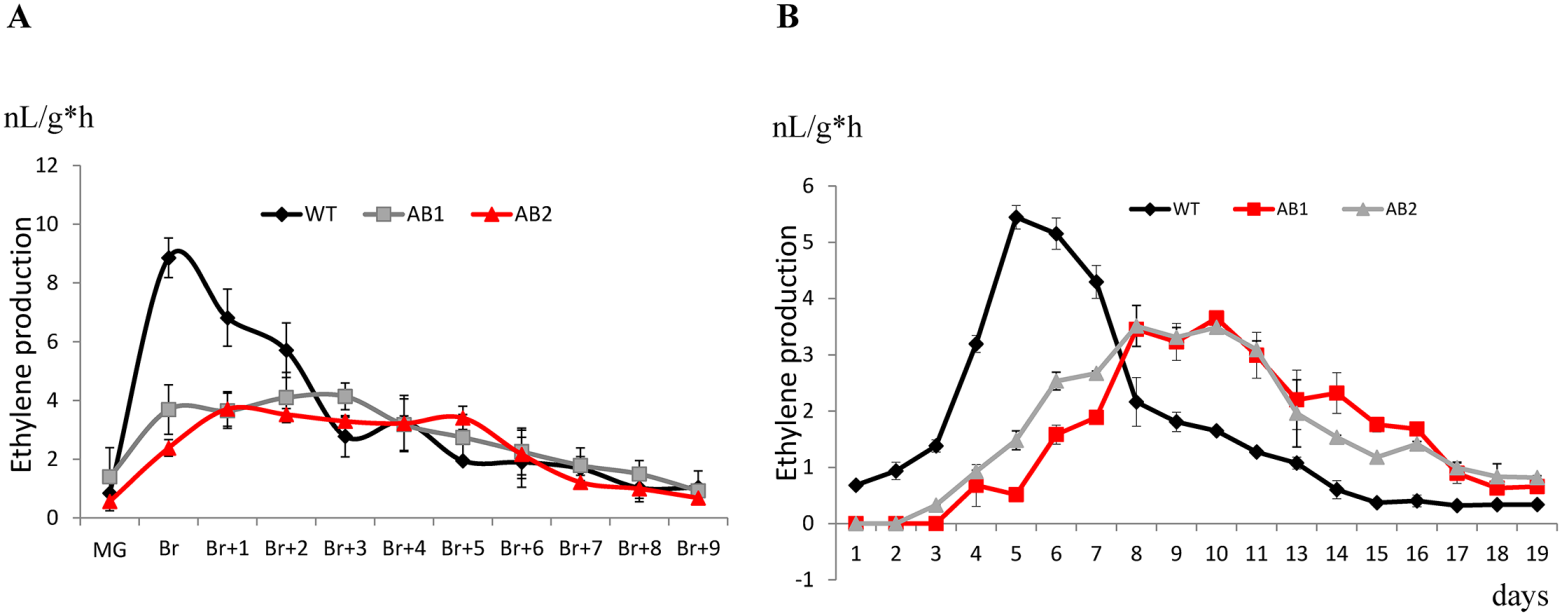
Ethylene production of *SlARF2AB-RNAi* fruits. (A) Ethylene production of wild-type and *SlARF2AB-RNAi* fruits picked at different ripening stages and assessed for ethylene production. MG = mature green stage; Br = breaker stage; Br+1 = 1 day post breaker stage; Br+2 = 2 days post breaker stage; Br+3, 3 days post breaker stage. (B) Ethylene production of wild-type and *SlARF2AB-RNAi* fruits picked at MG stage and left on the bench. Ethylene was measured at different days post mature green stage. Values represent means of at least 10 individual fruits. Vertical bars represent SD. AB1 = *SlARF2AB-RNAi* line 311; AB2 = *SlARF2AB-RNAi* line 223.

**Fig 8:**
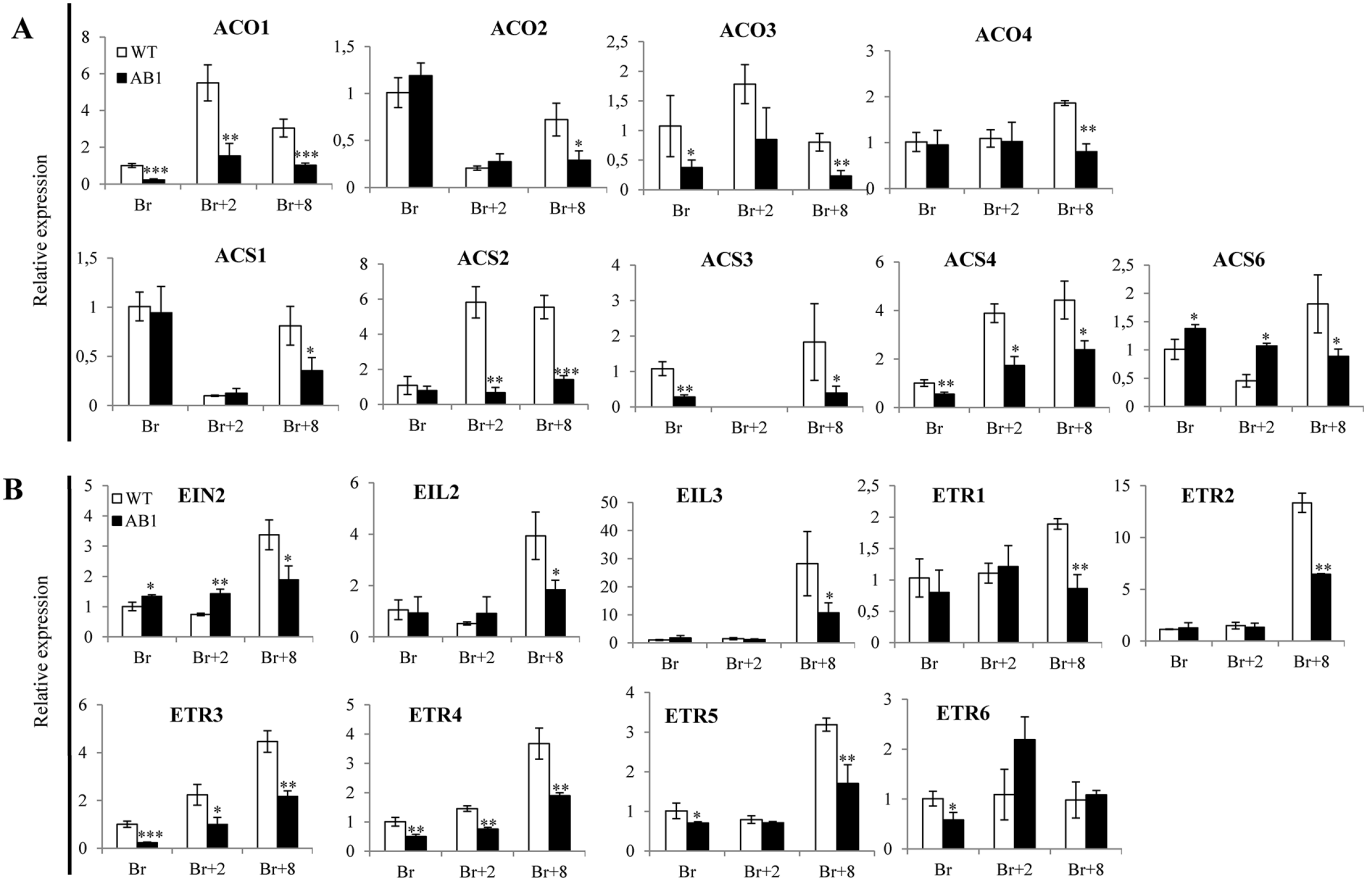
The expression of ethylene synthesis and ethylene perception genes is altered in *SlARF2AB-RNAi* plants. (A) Expression of ethylene synthesis pathway genes in *SlARF2AB-RNAi* lines assed by Quantitative RT-PCR. *ACO1*, *ACO2*, *ACO3*, *ACO4* aminocyclopropane-1-carboxylic acid oxidase; *ACS1*, *ACS2*, *ACS3*, *ACS4*, *ACS6* aminocyclopropane-1-carboxylic acid synthases. (B) Expression of ethylene perception genes in *SlARF2AB-RNAi* assessed by Quantitative RT-PCR. EIN2 ethylene signaling protein; EIL2 and EIL3 are EIN3-like proteins; ETR1, ETR2, ETR3 (NR, never-ripe), ETR4, ETR5, ETR6 ethylene receptors; CTR1 ethylene-responsive protein kinase. ABL1 refers to *SlARF2AB-RNAi* line 311. Total RNA was extracted from different fruit developmental stages (breaker, Br; Br+2, 2 days post-breaker; Br+8, 8 days post-breaker). The relative mRNA levels of each gene in WT at the breaker (Br) stage were standardized to 1.0, referring to the *SlActin* gene as internal control. Error bars mean ±SD of three biological replicates. Stars indicate statistical significance using Student’s t-test: * p-value<0.05, ** p-value<0.01.

**Fig 9:**
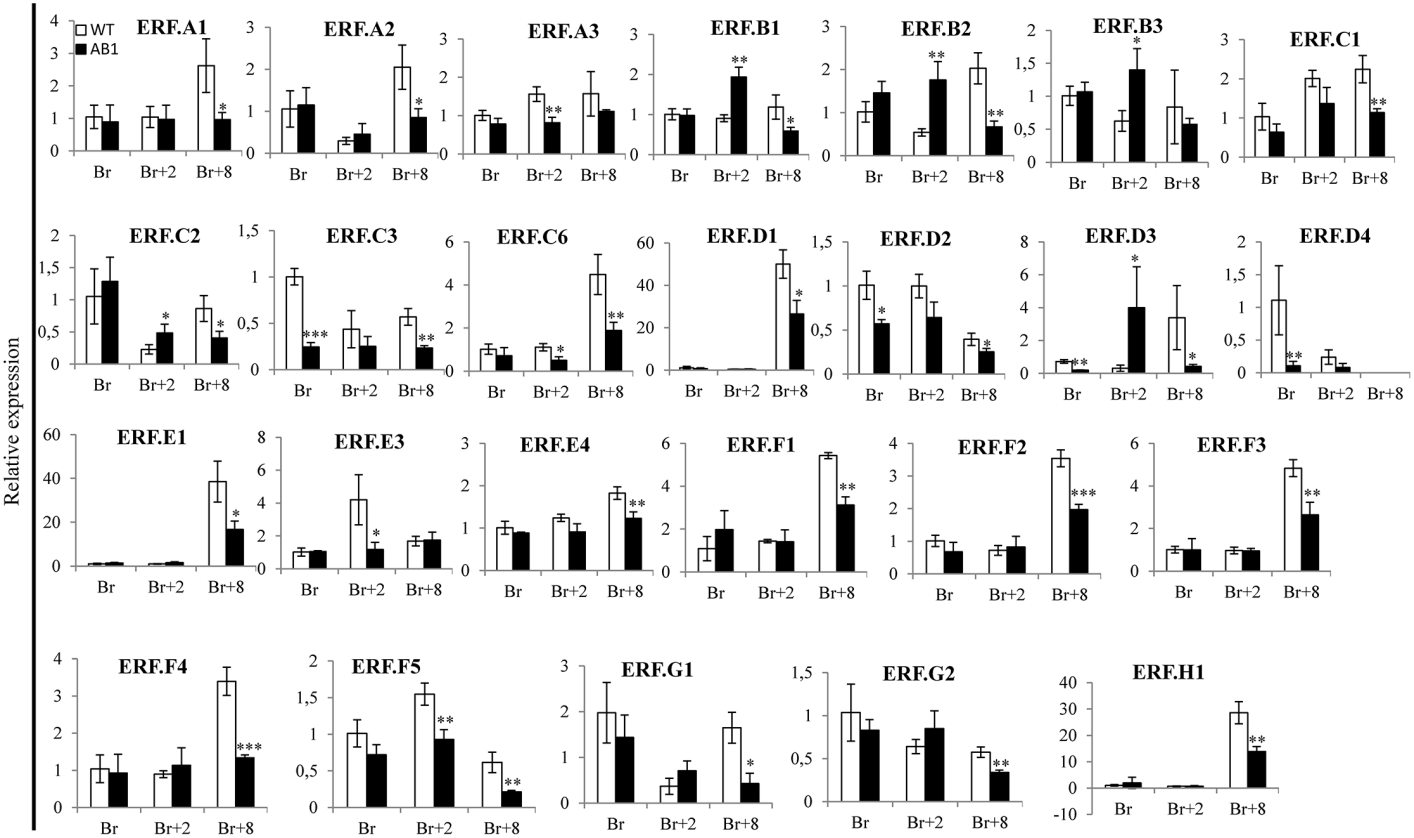
The expression of ERF genes in wild type and *SlARF2AB-RNAi* plants. Expression of ERF family genes in *SlARF2AB-RNAi* fruits assessed by Quantitative RT-PCR. Total RNA was extracted from different fruit developmental stages (breaker, Br; Br+2, 2 days post-breaker; Br+8, 8 days post-breaker). The relative mRNA levels of each gene in WT at the breaker (Br) stage were standardized to 1.0, referring to the *SlActin* gene as internal control. Error bar means ±SD of three biological replicates. Stars indicate statistical significance using Student’s t-test: * p-value<0.05, ** p-value<0.01. ABL1 refers to *SlARF2AB-RNAi* line 311.

The ripening defect phenotype prompted us to monitor the climacteric ethylene production in the *SlARF2AB-RNAi* line. Ethylene production, assessed either on fruits kept on the plant or detached (Fig 7), is significantly low throughout ripening and reaches its peak with 3 days delay as compared to wild type (Fig 7). Assessing the expression of ethylene biosynthesis genes by qPCR (Fig 8A) revealed reduced levels of *ACO1*, *ACS2*, *ACS3* and *ACS4* transcripts in the *SlARF2AB RNAi* line at all ripening stages (Breaker, Breaker+2 and Breaker+8). However, the reduced ethylene production cannot account for the ripening defects because exogenous ethylene treatment failed to reverse the ripening phenotype (Fig 6D). We therefore examined the expression of ethylene receptor genes (Fig 8B). transcript levels corresponding to *ETR3* (*NR*) and *ETR4* are dramatically low in the transgenic lines compared to wild type at all stages of fruit ripening (Br, Br+2, and Br+8) and the expression of other receptor genes (*ETR1*, *ETR2*, and *ETR5*) is also down-regulated at the breaker+8 stage. The disturbed expression of ethylene receptor genes is likely to result in altered ethylene perception in the transgenic lines. In addition, the expression of *EIN2* and two EIN3-like genes (*EIL2* and *EIL3*), which encode major components of ethylene transduction pathways, was also down-regulated during ripening of *SlARF2A/B RNAi* fruit (Fig 8B). More striking, the expression of a high number of *ERF* genes (Fig 9), known to mediate ethylene responses, was also altered with *SlERF*.*A1*, *SlERF*.*A2*, *SlERF*.*A3*, *SlERF*.*C1*, *SlERF*.*C3*, *SlERF*.*C6*, *SlERF*.*D1*, *SlERF*.*D2*, *SlERF*.*D4*, *SlERF*.*E1*, *SlERF*.*E3* and *SlERF*.*E4* being down-regulated while *SlERF*.*B1*, *SlERF*.*B2*, *SlERF*.*B3*, *SlERF*.*D3*, *SlERF*.*F2* are up-regulated. Altogether, these data strongly suggest that ethylene responses are highly impaired in the transgenic lines.

### *SlARF2AB-RNAi* fruits show reduced pigment accumulation and enhanced firmness

**Fig 10:**
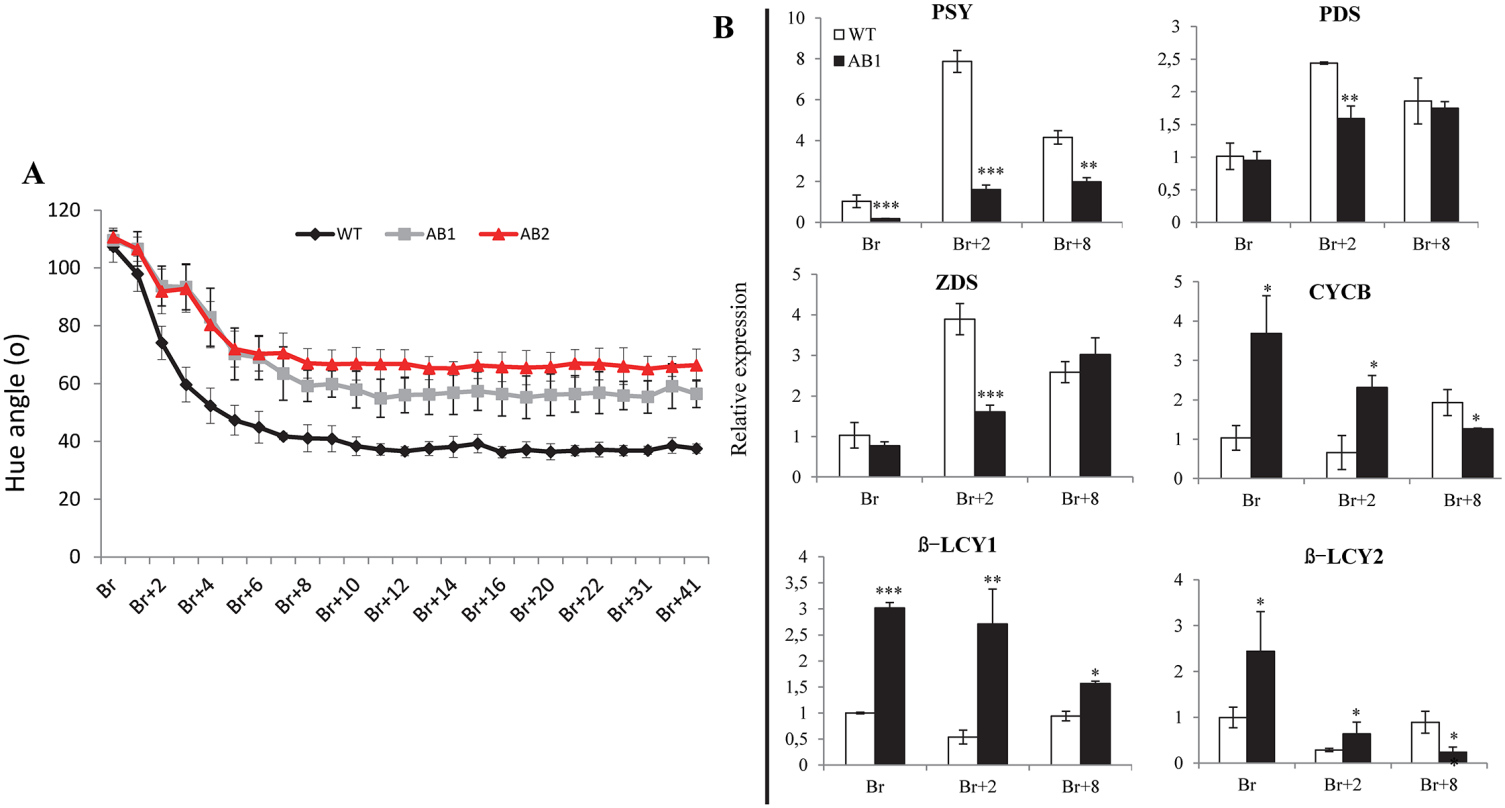
Altered pigment accumulation in *SlARF2AB-RNAi* fruits. (A) Changes in hue angle in WT and two independent *SlARF2AB-RNAi* lines during different ripening stages (breaker, Br; Br+1 to 41 days post-breaker;). AB1 = *SlARF2AB-RNAi* line 311; AB2 = *SlARF2AB-RNAi* line 223. (B) Quantitative RT-PCR relative expression of carotenoid biosynthesis genes in wild-type (WT) and *SlARF2AB-RNAi* tomato lines. Total RNA was extracted from different developmental stages of fruit (breaker, Br; Br+2, 2 days post-breaker; Br+8, 8 days post-breaker). The relative mRNA levels of each gene in WT at breaker (Br) stage were standardized to 1.0, referring to the *SlActin* gene as internal control. Error bar means ±SD of three biological replicates. Stars indicate a statistical significance using Student’s t-test: * p-value<0.05, ** p-value<0.01. ABL1 is *SlARF2AB-RNAi* line 311. PSY1 phytoene synthase; PDS phytoene desaturase; ZDS, carotenoid desaturase; ß-LCY1, ß-LCY2, CYC-ß lycopene b-cyclases.

**Fig 11:**
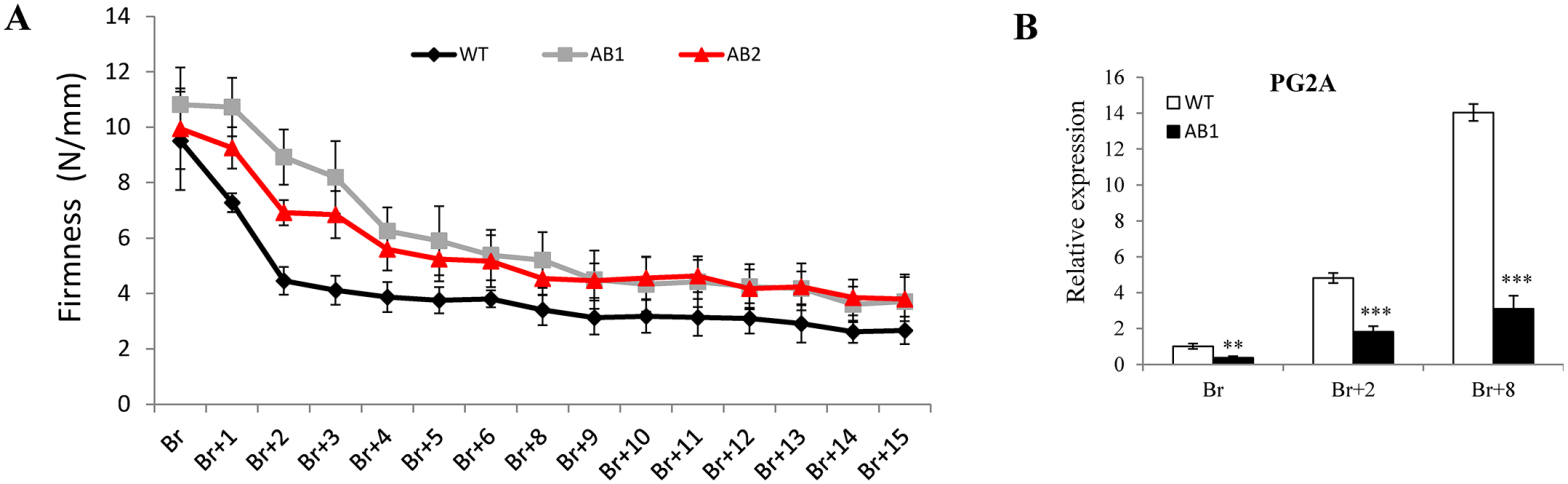
Altered firmness in *SlARF2AB-RNAi* fruits. (A) Firmness of wild-type and *SlARF2AB-RNAi* fruits. Fruits were harvested at breaker stage, kept at room temperate and firmness was measured day by day. A total of 15 fruits were used for each measurement and the error bars represent ±SD. AB1 = *SlARF2AB-RNAi* line 311; AB2 = *SlARF2AB-RNAi* line 223. (B) Quantitative RT-PCR relative expression of polygalacturonase gene *PG2A* at different ripening stages in *SlARF2AB-RNAi* and wild type fruits (breaker, Br; Br+2, 2 d post-breaker; Br+8, 8 d post-breaker). Relative mRNA levels in WT at the breaker (Br) stage were standardized to 1.0, referring to *SlActin* gene as internal control. Error bars represent ±SD of three biological replicates. Stars indicate a statistical significance using Student’s t-test: * p-value<0.05, ** p-value<0.01. ABL1 is *SlARF2AB-RNAi* line 311.

The fruit color saturation assessed by Hue angle, indicative of color intensity, revealed a reduced red pigment accumulation in *SlARF2AB* down-regulated lines (Fig 10). Accordingly, the expression of genes involved in the carotenoid pathway was altered. *PSY1*, a key regulator of flux through the carotenoid pathway, was significantly down-regulated in the *SlARF2AB-RNAi* fruits at all ripening stages (Fig 10). Lower levels of phytoene desaturase (PDS) and phytoene synthase (ZDS) transcripts were also observed at Br+2 stage in the *SlARF2AB-RNAi* fruit. By contrast, transcripts corresponding to lycopene beta cyclase genes (*β-LCY1*, *β-LCY2*) displayed higher accumulation than in wild-type at all ripening stages, and those corresponding to lycopene β-cyclases (*CYCB*) were also up-regulated at Br and Br+2 stages in *SlARF2AB-RNAi* fruit (Fig 10). On the other hand, *SlARF2AB-RNAi* fruits maintained higher firmness than wild type throughout ripening (Fig 11). In line with this delayed softening phenotype, transcript accumulation of *PG2A*, a major fruit polygalacturonase gene involved in ripening-related cell wall metabolism, was significantly reduced at Br, Br+2, and Br+8 stages in *SlARF2AB-RNAi* fruits (Fig 11).

### Expression of ripening regulator genes is altered in *SlARF2* down-regulated lines

**Fig 12:**
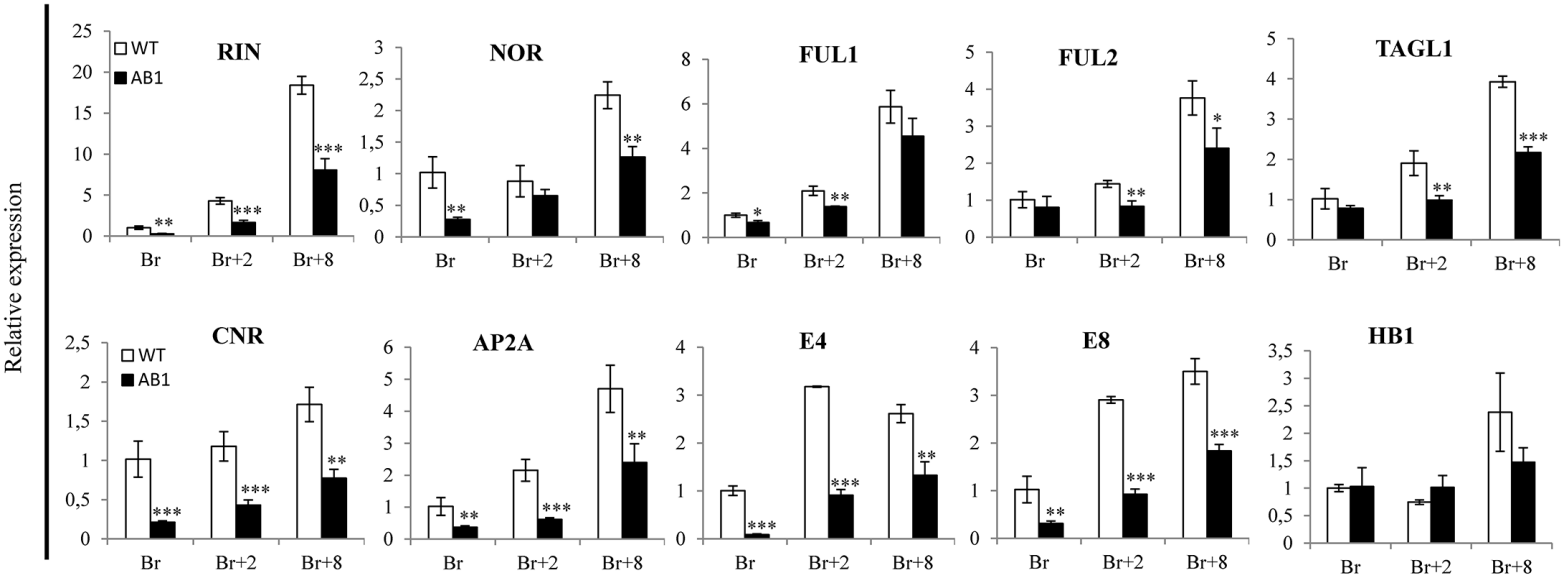
The expression of a number of ripening-related genes is altered in *SlARF2AB-RNAi* plants. Quantitative RT-PCR relative expression of ripening regulator genes in wild-type (WT) and *SlARF2AB-RNAi* lines during fruit ripening. Total RNA was extracted from the indicated developmental stages of fruit (breaker, Br; Br+2, 2 days post-breaker; Br+8, 8 days post-breaker). The relative mRNA levels of each gene in WT at the breaker (Br) stage were standardized to 1.0, referring to the *SlActin* gene as internal control. Error bar means ±SD of three biological replicates. Stars indicate statistical significance using Student’s t-test: * p-value<0.05, ** p-value<0.01. AP2a, APETALA2/ERF gene; CNR, colorless non-ripening; HB-1, HD-Zip homeobox; NOR, non-ripening; RIN, ripening inhibitor; TAGL1, tomato AGAMOUS-LIKE 1. FUL1, FUL2 MADS domain transcription factors; E4, E8 ethylene-responsive and ripening-regulated genes.

The expression of key ripening regulators assessed at the transcript level was strongly reduced throughout ripening in the *SlARF2 RNAi* line. Compared to wild type fruit, transcript levels corresponding to *RIN* and *CNR* genes were significantly lower at Br, Br+2 and Br+8 stages (Fig 12). Likewise, the *NOR* gene displayed reduced expression at Br and Br+8 stages, *TAGL1* showed the same tendency at Br and Br+2 stages, *FUL1* at Br and Br+2 stages, and *FUL2* at Br+2 and Br+8 stages. The altered expression of these genes is consistent with the dramatically altered ripening of *SlARF2AB-RNAi* fruits. Likewise, the low expression level of *E8* and *E4*, two ethylene-responsive and ripening- associated genes, is consistent with the altered expression of ethylene biosynthesis and signaling genes. By contrast, mRNA levels of *LeHB-1*, another ripening regulator gene, did not display significant change in *SlARF2AB-RNAi* fruits compared to wild type (Fig 12).

## Discussion

**Fig 13:**
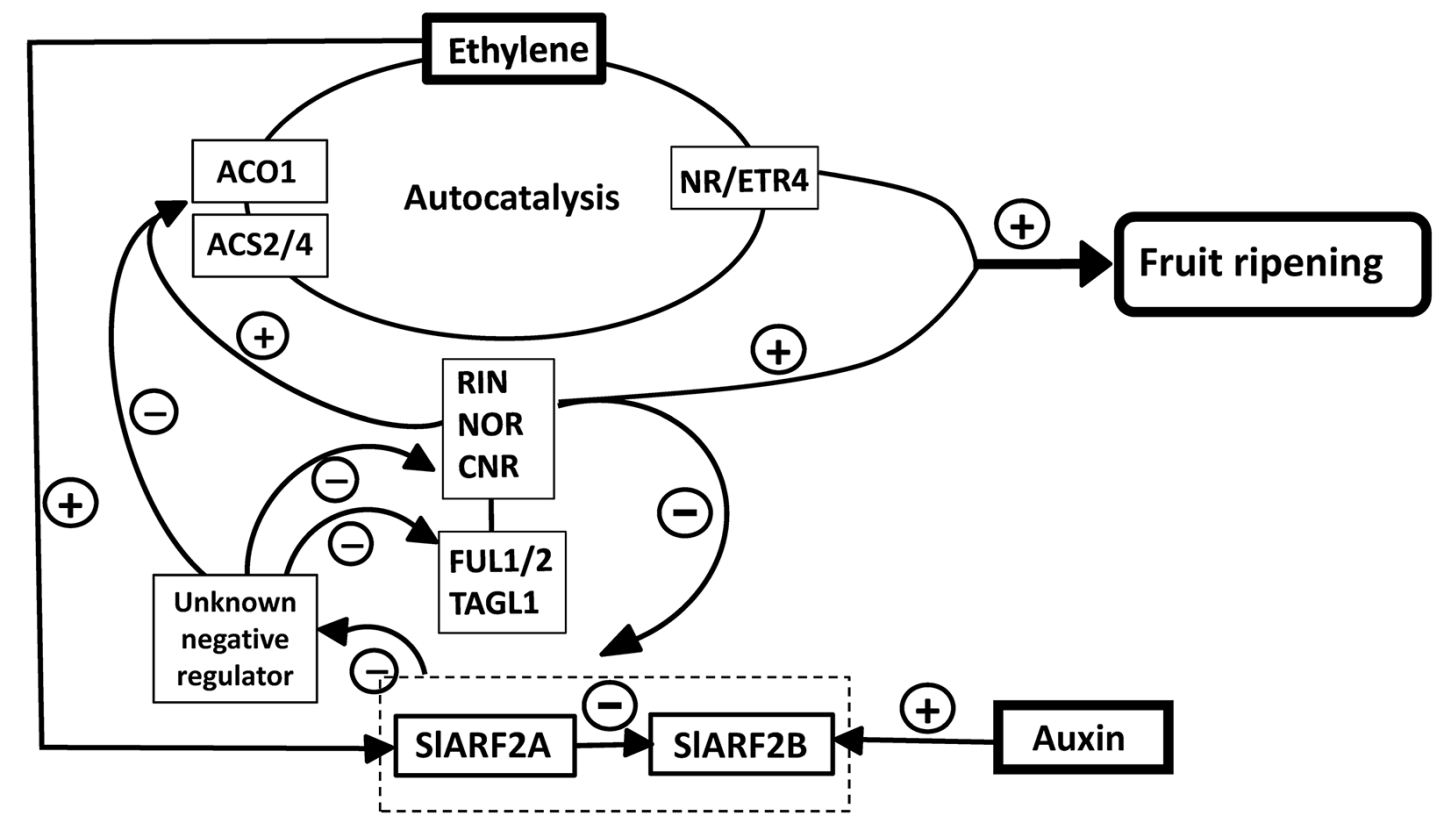
A synthetic model positioning SlARF2 in the regulatory network controlling fruit ripening. SlARF2 mediates tomato fruit ripening by positively regulating key ethylene biosynthesis genes (*ACO1*, *ACS2/4*) and through modulating key regulators of fruit ripening such as RIN, NOR, and CNR transcription factors known to affect ripening by positively regulating *ACO1* and *ACS2/4*. *SlARF2A* is up-regulated by ethylene while *SlARF2B* is up-regulated by auxin. SlARF2A negatively regulates the expression of SlARF2B, thus down-regulation of SlARF2A is compensated by an up-regulation of SlARF2B. SlARF2 also modulates the expression of *FUL1/2* and *TAGL1*. It is postulated that SlARF2 negatively regulates at the transcription or at the protein level an unknown factor that acts as a ripening repressor.

While ethylene is considered as the key hormone regulating climacteric fruit ripening, the down-regulation of *SlARF2* described herein supports the idea that auxin might also play an important role in the control of the ripening process. The altered ripening phenotypes associated with the under-expression of *SlARF2* genes are consistent with previous work showing that the coordinated expression of some ARF genes in the tomato is instrumental to normal fruit ripening [28,32,40]. As depicted in the model proposed (Fig 13), besides the crucial role devoted to ethylene, the data support a higher order of complexity of the mechanism underlying the control of fleshy fruit ripening which should be rather seen as a multi-hormonal process. Molecular analyses indicate that SlARF2 impacts, either directly or/and indirectly, the expression of master regulators of ripening like RIN, NOR and CNR and components of ethylene biosynthesis and responses (Fig 13). The data clearly support the idea of SlARF2 being a major component of the regulatory mechanism controlling tomato fruit ripening. It remains however unclear how the knockdown of a transcriptional repressor leads to the down-regulation of a set of genes whose expression is instrumental to climacteric ripening. Given that SlARF2 works as transcriptional repressors, the data imply that their main target could be a negative regulator of the ripening process. While the nature of this putative negative regulator remains to be elucidated, the data indicate that this unknown component has the ability to regulate the key factors controlling fruit ripening such as *MADS-Box* and ethylene signaling genes. SlARF2 genes are obviously required for climacteric ripening, hence the hypothesis that the rise of their expression at the onset of ripening may inhibit a negative regulator either at the transcriptional or the protein level, thus releasing the expression of key ripening genes (Fig 13). However, despite their repressor activity on auxin-responsive promoters, it cannot be fully excluded that ARF2A/B may also have the ability to function as activator on the promoter of key genes regulating fruit ripening, such as *Rin* and *Nor*.

While the expression of *SlARF2A* and *SlARF2B* increases during fruit ripening, SlARF2A also displays a high expression level in leaves and flowers suggesting an active role for this gene in vegetative organs. Single knockdown of either *SlARF2A* or *SlARF2B* resulted in discreet ripening phenotypes, whereas simultaneous down-regulation of the two genes leads to a severe delay or almost complete inhibition of ripening, indicating that both genes may contribute to tomato fruit ripening. Genetic crosses between *SlARF2 RNAi* tomato lines and lines expressing the GUS reporter driven by the DR5 synthetic auxin-responsive promoter indicated that single repression of *SlARF2A* or *SlARF2B* is unable to significantly affect *GUS* expression while simultaneous down-regulation of both *SlARF2* genes resulted in a strong increase in DR5:GUS expression similar to that observed upon exogenous auxin treatment (Fig 5). These data indicate that, *in planta*, SlARF2 acts as a repressor of auxin-dependent gene transcription and clearly suggests that SlARF2A and SlARF2B are functionally redundant. Moreover, down-regulation of *SlARF2A* is compensated by an up-regulation of *SlARF2B* suggesting a coordinated expression of the two *ARF* paralogs. Indeed, transient expression assay revealed the ability of SlARF2A to repress the activity of *SlARF2B* promoter indicating that the transcription of this latter gene is under direct regulation by SlARF2A.

Down-regulation of *SlARF2* genes impairs normal fruit ripening likely via altering components of ethylene metabolism, signaling and response. In support of this idea, *SlARF2A/B RNAi* fruits produce less climacteric ethylene than wild type (Fig 7) and lower expression of ACC oxidase (*ACO*) and ACC synthase (*ACS*) genes whose expression is instrumental to the triggering of climacteric ripening [5,9]. It was shown that transition from auto-inhibitory System1 to auto-catalytic System2 is associated with an increased expression of *LeACS1A*, *LeACS2*, *LeACS4*, *LeACO1*, *LeACO3*, and *LeACO4* genes [5,9,42]. Accordingly, repression of genes belonging to *ACS* and *ACO* gene families blocked fruit ripening in tomato [6,7,9,43]. In line with the reduced ethylene production in the *SlARF2AB-RNAi* fruits, the expression of ethylene responsive genes *E4* and *E8* is also reduced (Fig 12). The treatment with exogenous ethylene was unable to restore normal fruit ripening suggesting that ethylene signaling is likely impaired in *SlARF2* knockdown lines. The expression of ethylene receptor genes *NR* (*SlETR3*), *SlETR4*, and *SlETR6* is altered in the transgenic lines which may account for the loss of ability to trigger the autocatalytic ethylene production required for normal climacteric ripening even upon exogenous ethylene treatment. It was reported that down-regulation of NR receptor resulted in slight delay in fruit ripening with reduced rates of ethylene synthesis and slower carotenoid accumulation [44]. However, reducing *NR* expression via RNA antisense strategy has been also reported to result in up-regulation of LeETR4 as a compensation mechanism for the loss of NR [44]. In the *SlARF2* under-expressing fruit, both *SlETR3/NR* and *SlETR4* were down-regulated (Fig 8), which may explain the more severe loss of fruit ripening in *SlARF2AB-RNAi* lines compared to *NR* antisense lines. It is widely accepted that modulation of the expression of ethylene-regulated genes is at least partly mediated by ERFs [20,45–49]. In particular, it was shown that SlAP2a, a tomato *APETALA2/ERF* gene, is a negative regulator of fruit ripening [50,51] and that SlERF6 plays an important role in tomato ripening and carotenoid accumulation [48]. More recently, the expression of a dominant repression version of another tomato ERF gene, *SlERF*.*B3*, was shown to lead to a dramatic delay in fruit ripening [52]. Interestingly, the expression of a high number of ERFs is disturbed in *SlARF2AB-RNAi* fruits which may account for the altered ethylene response and contribute to the ripening defect phenotypes.

Tomato genes encoding ripening-inhibitor (RIN), non-ripening (NOR) and colorless non-ripening (CNR) are considered as master regulators of the ripening process and mutations in the corresponding loci dramatically impair fruit ripening [15,19,21]. Some of the main features of these non-ripening mutants are shared by the *SlARF2* knockdown lines such as enhanced fruit firmness, low ethylene production and incapacity to ripen in response to exogenous ethylene. Moreover, the expression of *RIN*, *NOR* and *CNR* genes was significantly down-regulated during fruit ripening in *SlARF2AB-RNAi* lines (Fig 12). Considering the crucial role of *RIN*, *NOR*, and *CNR* in the attainment of competence to ripen [53], the down-regulation of these master transcriptional regulators in *SlARF2* under-expressing fruits is likely contributing to the impaired ripening phenotype. *SlARF2AB-RNAi* fruits showed yellow-orange color associated with a reduced expression of *AGAMOUS-like 1* (*TAGL1*) and *FRUITFUL FUL1* and *FUL2* orthologs encoding ripening-related MADS domain transcription factors. Accordingly, suppression of *TAGL1* was shown to result in yellow-orange fruits and low ethylene levels due to the down-regulation of *ACS2* [17,18]. Likewise, simultaneous suppression of *FUL1* and *FUL2* resulted in ripening defects [54]. Strikingly, these phenotypes are similar to those displayed by SlARF2 down-regulated lines. It has been reported that TAGL1, FUL1, and FUL2 interact with RIN [55,56] forming higher order complexes that regulate tomato fruit ripening [57]. The phenotypes and associated gene expression patterns support the hypothesis that down-regulation of SlARF2 impairs ripening through interfering with the MADS-box regulatory network. This work shows that the expression of SlARF2 is down-regulated in the tomato ripening mutants *rin* and *nor*, thus suggesting that these ripening regulators negatively regulate the expression of *SlARF2* genes. Taken together, the data support the hypothesis of an active interplay between the major ripening regulators, *rin* and *nor*, and SlARF2 which therefore emerges as a new player of the control mechanism of tomato fruit ripening (Fig 13).

It has been suggested that tomato SlARF2 might be involved in auxin and ethylene interplay during the apical hook formation [58,59]. This putative role in linking the two hormones signaling is in agreement with the presence of conserved auxin and ethylene-responsive elements in the promoter regions of *SlARF2A* and *SlARF2B*. Down-regulation of SlARF2 leads to altered expression of transcription factors known to mediate both ethylene (ERFs) and auxin (ARFs) responses and results in disturbed expression of auxin and ethylene responsive genes further suggesting the potential involvement of SlARF2A and SlARF2B in the crosstalk between auxin and ethylene.

A typical feature of tomato fruit undergoing ripening is the accumulation of lycopene which accounts for the red color whereas β-carotene, conferring an orange color, does not accumulate normally at this stage [60,61]. The *SlARF2AB-RNAi* fruit displayed yellow-orange sectors reflecting increased accumulation of β-carotene and degraded lycopene. The accumulation of lycopene is caused by the up-regulation of the phytoene synthase gene (*PSY1*) and the down-regulation of *LCYB* and *CYCB* [60,62–64]. PSY1 is the first rate-limiting enzyme in the plant carotenoid biosynthetic pathway and its transcript accumulation is induced by ethylene [60,65]. Repression of *PSY1* inhibits total carotenoid accumulation resulting in mature yellow fruit with little lycopene or βcarotene [65]. LCYB and CYCB are responsible for the conversion of lycopene into β-carotene, which turns the fruit orange [61,63]. During fruit ripening, transcript accumulations of both genes is repressed by the elevated ethylene levels thus leading to the accumulation of lycopene that is responsible for the red color of ripe fruit [18]. The *SlARF2AB-RNAi* fruit produced less ethylene than wild type and exhibited low levels of *SlPSY1* transcripts and high levels of *SlLCYB* and *SlCYCB*, which promotes the accumulation of β-carotene rather than lycopene thus causing the orange-yellow color of *SlARF2AB-RNAi* fruit.

Overall, the work adds another layer to the gene regulatory network underlying fruit ripening reinforcing the concept that the ripening process relies on the interplay between different actors. While the present study is in line with previous reports [28,32,40] supporting the potential role of auxin in fleshy fruit ripening, there is little doubt that the involvement of other hormones is also likely required for a proper tuning of this complex developmental process. Altogether, the data sustain a high level of complexity of the signaling networks underlying fleshy fruit ripening which may reflect the diversity of the ripening features displayed by different plant species.

## Materials and Methods

### Plant materials and growth conditions

Tomato (*Solanum lycopersicum* L. *cv* MicroTom) seeds were sterilized, washed with sterile water 5 times, and sown in Magenta vessels containing 50ml of 50% Murashige and Skoog (MS) medium with 0.8% (w/v) agar, pH 5.9. The transgenic plants were transferred to soil and grown under standard greenhouse conditions [32]. Conditions in the culture chamber room were set as follows: 14-h-day/10-h-night cycle, 25/20°C day/night temperature, 80% relative humidity, 250 mol.m^-2^.s^-1^ intense light [52].

### Plant transformation

Three cDNA fragments specific to *SlARF2A*, *SlARF2B* and both were cloned into pHellsgate12 vector independently, with primers listed in the S1 Table. Transgenic plants were generated by Agrobacterium-mediated transformation [66] with minor changes: 6 days old cotyledons were used for the transformation; the duration of subcultures for shoot formation was reduced to 15 days; and the kanamycin concentration was 70 mg.L^-1^. The constructs were under the transcriptional control of the CaMV 35S and the Nos terminator [32].

### Sequence structure and promoter analysis

The structure of the *SlARF2A* and *SlARF2B* were determinated using *in silico* approaches (software: Fancy Gene V1.4). Protein domains were first predicted on the prosite protein database (http://prosite.expasy.org/). Promoter sequences of *SlARF2A* and *SlARF2B* genes were analyzed using PLACE signal scan search software (http://www.dna.affrc.go.jp/PLACE/signalscan.html).

### Flower emasculation and cross fertilization assays

Flower buds of *DR5*:*GUS* transgenic plants were emasculated before dehiscence of anthers (closed flowers) to avoid accidental self-pollination. Cross-pollination was performed on *DR5*:*GUS* emasculated flowers with pollen from wild type, *SlARF2A-RNAi*, *SlARF2B-RNAi*, and *SlARF2AB-RNAi* flowers.

### Subcellular localization of SlARF2A and SlARF2B

For localization of SlARF2A and SlARF2B proteins, the CDS sequences were cloned as a C-terminal fusion in frame with green fluorescent protein (GFP) into the pGreen-GFP vector, and expressed under the control of the 35S CaMV promoter. The pGreen-GFP empty vector was used as the control. Protoplasts were obtained from tobacco suspension-cultured (*Nicotiana tabacum*) BY-2-cells and transfected according to the method described previously [67]. GFP localization by confocal microscopy was performed as described previously [38].

### Transient expression using a single cell system

For co-transfection assays, the coding sequence of SlARF2A and SlARF2B were cloned into the pGreen vector and expressed under the control of the 35S CaMV promoter. The synthetic DR5 promoter containing AuxRE and the promoter of *SlARF2B* were cloned in frame with GFP reporter gene in pGreen vector independently. Protoplasts were obtained from suspension-cultured of tobacco (*Nicotiana tabacum*) BY-2-cells and transfected according to the method described previously [67]. After 16 h of incubation in the presence or absence of 2.4-D (50 μM), GFP expression was analyzed and quantified by flow cytometry (FACS Calibur II instrument, BD Biosciences, San Jose, CA, USA) as indicated in Hagenbeek and Rock (2001). All transient expression assays were repeated at least three times with similar results.

### GUS staining and analysis

To visualize GUS activity, transgenic lines bearing the promoter of *DR5* fused with GUS constructs were incubated with GUS staining solution (0.1% Triton X-Gluc, pH7.2, 10 mM EDTA) at 37°C overnight. After GUS staining, samples were decolorized using several washes of graded ethanol series [32].

### Auxin, ethylene and 1-MCP treatment

For auxin treatment on light grown seedlings, 21-day-old *DR5*::*GUS* seedlings were soaked in liquid MS medium with or without (mock treatment) 20 μM IAA for 2 hours. For auxin treatment on fruit, mature green fruits were injected with 20 μM IAA and kept for 6 hours at room temperature. For ethylene treatment on fruit, mature green fruits were treated with air or ethylene gas (50 μL.L^-1^) for 5 hours. For 1-MCP treatment, 1.0 mg.L^-1^ 1-MCP was applied into the breaker stage fruits for 16 hours. For qPCR expression analysis, the tissues were immediately frozen in liquid nitrogen and stored at -80°C until RNA extraction.

### Ethylene production and ethylene response

Fruits from different developmental stages were harvested and incubated in opened 125-ml jars for 3 hours to remove the wound ethylene production caused by picking. Jars were then sealed and incubated at room temperature for 2 hours, and 1 ml of headspace gas was injected into an Agilent 7820A gas chromatograph equipped with a flame ionization detector (Agilent, Santa Clara, CA, USA). Samples were compared to 1 ml.L^-1^ ethylene standard and normalized for fruit weight. For ethylene response assay, mature green fruits from wild-type and *SlARF2AB-RNAi* lines were treated by 10 ml.L^-1^ ethylene for 3 days, 2 hours and 3 times per day.

### Firmness measurement

Fifteen fruits from each line of the *SlARF2AB-RNAi* and wild type were harvested at the Breaker (Br) stage. The firmness was then assessed using Harpenden calipers (British Indicators Ltd, Burgess Hill, UK) as described by Ecarnot et al., (2013). After the first measurement, these fruits were kept at room temperature for measuring the firmness day by day.

### Color measurement

Twenty fruits for each line of the *SlARF2AB-RNAi* and wild type were harvested at the Br stage. The hue angle values were calculated according to the methods previously described [32]. After measurement, these fruit were kept at room temperature and were measured day by day until fruits got fully red.

### RNA extraction and quantitative RT-PCR

Different stage fruits were harvested, the pericarp were frozen in liquid nitrogen, stored at -80°C. Total RNA extraction, DNA contamination removing, cDNA generation of tomato tissues (root, stem, leaves, bud, flower, mature green fruit, breaker fruit, and red fruit) and qRT-PCR were performed according to methods previously described [38,68]. The primer sequences are listed in the S1 Table. Actin was used as the internal reference. Three independent RNA isolations were used for cDNA synthesis and each cDNA sample was subjected to real-time PCR analysis in triplicate.

### Accession number

The sequences of genes used for the qPCR can be found at the website (http://solgenomics.net/) under the following solyc numbers: *Sl-ERF*.*A1* (Solyc08g078180), *Sl-ERF*.*A2* (Solyc03g093610), *Sl-ERF*.*A3* (Solyc06g063070), *Sl-ERF*.*B1* (Solyc05g052040), *Sl-ERF*.*B2* (Solyc02g077360), *Sl-ERF*.*B3* (Solyc05g052030), *Sl-ERF*.*C1* (Solyc05g051200), *Sl-ERF*.*C2* (Solyc04g014530), *Sl-ERF*.*C3* (Solyc09g066360), *Sl-ERF*.*C6* (Solyc03g093560), *Sl-ERF*.*D1* (Solyc04g051360), *Sl-ERF*.*D2* (Solyc12g056590), *Sl-ERF*.*D3* (Solyc01g108240), *Sl-ERF*.*D4* (Solyc10g050970), *Sl-ERF*.*E1* (Solyc09g075420), *Sl-ERF*.*E2* (Solyc09g089930), *Sl-ERF*.*E3* (Solyc06g082590), *Sl-ERF*.*E4* (Solyc01g065980), *Sl-ERF*.*F1* (Solyc10g006130), *Sl-ERF*.*F2* (Solyc07g064890), *Sl-ERF*.*F3* (Solyc07g049490), *Sl-ERF*.*F4* (Solyc07g053740), *Sl-ERF*.*F5* (Solyc10g009110), *Sl-ERF*.*G1* (Solyc01g095500), *Sl-ERF*.*G2* (Solyc06g082590), *Sl-ERF*.*H1* (Solyc06g065820), *PSY1* (Solyc03g031860), *PDS* (Solyc03g123760), *ZDS* (Solyc01g097810), *β-LCY1* (Solyc04g040190), *β-LCY2* (Solyc10g079480), *CYC-β* (Solyc06g074240), *ACS2* (Solyc01g095080), *ACS4* (Solyc05g050010), *ACO1* (Solyc07g049530), *E4* (Solyc03g111720), *E8* (Solyc09g089580), *PG2a* (Solyc10g080210), *RIN* (Solyc05g012020), *CNR* (Solyc02g077850), *NOR* (Solyc10g006880), *HB1* (Solyc02g086930), *TAGL1* (Solyc07g055920), *AP2a* (Solyc03g044300), *EIN2* (Solyc09g007870), *EIL2* (Solyc01g009170), *EIL3* (Solyc01g096810), *ETR1* (Solyc12g011330), *ETR2* (Solyc07g056580), *ETR3* (NR) (Solyc09g075440), *ETR4* (Solyc06g053710), *ETR5* (Solyc11g006180), *ETR6* (Solyc09g089610), *CTR1* (Solyc10g083610), *ACS1* (Solyc08g081550), *ACS3* (Solyc02g091990), *ACS6* (Solyc08g008100), *FUL1* (Solyc06g069430), *FUL2* (Solyc03g114830), *ACO2* (Solyc12g005940), *ACO3* (Solyc07g049550), *ACO4* (Solyc02g081190). SAUR (Solyc09g007970.1.1), GH3 (Solyc01g107390.2.1), GUS (gb|KC920579.1|). The locus ID numbers of Sl-ARFs can be found in the publication of Zouine et al. (2014).

## Supporting Information

S1 Table List of primers used in the expression studies. (PDF)

S1 Fig Auxin related phenotypes displayed by SlARF2A/B-RNAi lines. (A) SlARF2A/B-RNAi lines showing the development of triple cotyledons. (B) SlARF2A/B lines showing root branching phenotypes.
(PDF)

S2 Fig Expression of *SlARF* in SlARF2AB-RNAi fruits assessed by Quantitative RT-PCR. Total RNA was extracted from WT and mutant fruits at the breaker stage. The relative mRNA levels of each *SlARF* gene in WT were standardized to 1.0, referring to the *SlActin* gene as internal control. Error bar means ±SD of three biological replicates. Stars indicate statistical significance using Student’s t-test: * p-value<0.05, AB1 refers to SlARF2AB-RNAi line 311.
(PDF)
