# Supplementary material for: Auxin Response Factor SlARF2 Is an Essential Component of the Regulatory Mechanism Controlling Fruit Ripening in Tomato
Source: PLoS Genet. 2015 Dec 30;11(12):e1005649. doi: 10.1371/journal.pgen.1005649 (PMC4696797; doi:10.1371/journal.pgen.1005649)
Supplement: S1 Table — (PDF) [file pgen.1005649.s001.pdf]

S1 Table List of primers used in the studies

Gene Name	Primer Sequence
SlARF2A RNAi	F 5'-aaaaagcaggcttc CTCCTCCCATGGGTTCTGTTTAC-3'
	F 5'-caagaaagctgggtc GGCTTTCCCAATAAGCTAAAATCC-3'
SlARF2B RNAi	F 5'-aaaaagcaggcttc GGGGCAATCTAGTTCATCTCATG-3'
	F 5'-caagaaagctgggtc TGTTTTGCCAGTAAGCTGAAATCAG-3'
SlARF2AB RNAi	F 5'-aaaaagcaggcttc TTCAGGCATATATTCCGGGGC-3'
	F 5'-caagaaagctgggtc GTTTTTGTTTGAATGGCATGCC-3'
Sl-Actin	F 5'-TGTCCCTATTTACGAGGGTTATGC-3'
	R 5'-CAGTTAAATCACGACCAGCAAGAT-3'
SlARF2A	F 5'-GCAAGGTCAAGAGTTATCGA-3'
	R 5'-CATTGGTTTCTGAGACAAGTC-3'
SlARF2B	F 5'-TTTAACGAGTATCCAACCTTCC-3'
	R 5'-GGGTTTAGGCATAATTTCTCCA-3'
E4	F 5'-GACCACTCTAAATCGCCAGG-3'
	R 5'-TTCCTGAGCGGTATTGCTTT-3'
E8	F 5'-TGGCTCCGAATCCTCCCAGTCT-3'
	R 5'-GTCCGCCTCTGCCACTGAGC-3'
SAUR	F 5'-AGACAACACCACTGTTCTTTC-3'
	R 5'-GCCTTGAGAAACTTACTGTCC-3'
GH3	F-5'AGCTCGTCATCACAACATACGC-3'
	R-5'GTTTATCGACAAGGGCGAGTTG-3'
GUS-1	F 5'-TACCGTACCTCGCATTACCC-3'
	R 5'-GCAGCAGTTTCATCAATCACC-3'
GUS-2	F 5'-ACCGATACCATCAGCGATCTC-3'
	R 5'-GTACCTTCTCTGCCGTTTCC-3'
ACO1	F 5'-GCCAAAGAGCCAAGATTTGA-3'
	R 5'-TTTTTAATTGAATTGGGATCTAAGC-3'

ACO2	F 5'-TTTATTACAAAGTGTGCGTCCCTA-3'
	R 5'-CTCATTTTTGGGTATTAAAATATGTGT-3'
ACO3	F 5'- GGAGCCTAGGTTTGAAGCAA-3'
	R 5'- AAACAAATTCCCCCTTGAAAA-3'
ACO4	F 5'- TGATCAAATTGCAAGTGCTTAAA-3'
	R 5'- ACCACACAACAATCACACACA-3'
ACS1	F 5'-TCGTTTCGAAGATTGGATGA-3'
	R 5'-CAACAACAACAAATCTAAGCCATT-3'
ACS2	F 5'-TGTTAGCGTATGTATTGACAACTGG-3'
	R 5'-TCATAACATAACTTCACTTTTGCATTC-3'
ACS3	F 5'-CCCTTGTCCACAAATCCAGA-3'
	R 5'-ACAGAGTGCACCCTCTAACATTT-3'
ACS4	F 5'-CTCCTCAAATGGGGAGTACG-3'
	R 5'-TTTTGTTTGCTCGCACTACG-3'
ACS6	F 5'-CTCCTATGGTCCAAGCAAGG-3'
	R 5'-CGACATGTCCATAATTGAACG-3'
EIN2	F 5'-GTGTGCTGAATAAGTTTAGTGGAG-3'
	R 5'-TGCTGTACAATAGAAGAATGGAGG-3'
EIL2	F 5'-TGAAGATGATGGAAGTCTGTAAGG-3'
	R 5'-CCACTCCCTGAGATTATCCGA-3'
EIL3	F 5'-ACAGGACTTCAAGAAACAACCA-3'
	R 5'-GTGTTGTGCTCATAGTTGATCTG-3'
ETR1	F 5'-GGAAGAACATTGGCATTGGAAG-3'
	R 5'-CCAACTGGATTTTGGTGTCGT-3'
ETR2	F 5'-TTGGAGGAATCAATGAGGGC-3'
	R 5'-TCATTACGCGCACGAACAG-3'
NR	F 5'-TGCTGTTCGTGTACCGCTTT-3'
	R 5'-TCATCGGGAGAACCAGAACC-3'
ETR4	R 5'-TGGAGGAGTGAGTGTGGATGC-3'

	F 5'-ATGGCTGTCGTTCTTGGGC-3'
ETR5	F 5'-GTGCTCTGGGCCCTTCACTA-3'
	R 5'-GAACTTACGCACCCTCAATGC-3'
ETR6	F 5'-TCAAAAAGCCGGTGATCTCG-3'
	R 5'-GCACCCATTTGAACGGAAAA-3'
CTR1	F 5'-CGATTTGAACATGACAGGGAG-3'
	R 5'-AAGGGATTGAGATGGAAGATGG-3'
Sl-ERF.A1	F 5'-ACCGGATCCTGTTAGAGTTGGA-3'
	R 5'-CGACGCCGATGAACAATG-3'
Sl-ERF.A2	F 5'-CGGTATCATCAGCTTCGGAAA-3'
	R 5'-TCTCAACTTCTAATTCGGCTTGCT-3'
Sl-ERF.A3	F 5'-GCGAAATGGATCAACAGTTACCA-3'
	R 5'-ATTAGACGACTGAAGCTTGAATTCC-3'
Sl-ERF.B1	F 5'-GAATGATGACGGAATTGTAATGAAGA-3'
	R 5'-TTCCACAATCCCAAATTGAAGA-3'
Sl-ERF.B2	F 5'-AGTTTGCAGCGGAGATTCGT-3'
	R 5'-TGCCCTGTCATATGCCTTTG-3'
Sl-ERF.B3	F 5'-CGGAGATAAGAGATCCAAGTCGAA-3'
	R 5'-CTTAAACGCTGCACAATCATAAGC-3'
Sl-ERF.C1	F 5'-TTCTTCGTGTCGAAAATACTAAGTTCAGT-3'
	R 5'-ACTCTAAATTCTTCAAGAAATCCAGAACA-3'
Sl-ERF.C2	F 5'-ATCATTACCATGGAATGATCAACATT-3'
	R 5'-CCGTCTATAACTTTCTTTCGAGGTTAA-3'
Sl-ERF.C3	F 5'-CAAGAAGTTTCCTCAATCTCTCATGTAT-3'
	R 5'-CCGAGATGAATAATCCATTTGATTT-3'
Sl-ERF.C6	F 5'-GGGAAATACGCTGCGGAAA-3'
	R 5'-TTTCGAACGTACCTAGCCATACTCT-3'
Sl-ERF.D1	F 5'-GGCAGCTGAAATAAGAGATCCATATAA-3'
	R 5'-CTAGCAGCCCCTTCAGCAGTAT-3'

Sl-ERF.D2	F 5'-ACACAAGTAGCACCAGCACCACTA-3'
	R 5'-ACCCCAAAAAAAGCAAGAAAATT-3'
Sl-ERF.D3	F 5'-ATTCATTTTCGGGTTGTGCAGTA-3'
	R 5'-CGACTATAATGATTTCTGCCGAACT-3'
Sl-ERF.D4	F 5'-GTTGCTGCTTTAACCAATGTGATTAT-3'
	R 5'-CTTCCGGTACGCGAAACAAG-3'
Sl-ERF.E1	F 5'-GTTCCTCTCAACCCCAAACG-3'
	R 5'-TTCATCTGCTCACCACCTGTAGA-3'
Sl-ERF.E2	F 5'-ACTTCGTGAGGAAACCCTGAAC-3'
	R 5'-GTTACTAATATAAGTCATGTTGGGCTGAA-3'
Sl-ERF.E3	F 5'-GCATTTGCGATCTGAAGTTGTT-3'
	R 5'-CAAATGGCTTGACATCGACTTG-3'
Sl-ERF.E4	F 5'-AGGCCAAGGAAGAACAAGTACAGA-3'
	R 5'-CCAAGCCAAACGCGTACAC-3'
Sl-ERF.F1	F 5'-ACGAGCTTTCTTCTTTTCTCTCTCTAAA-3'
	R 5'-GAAACTCGATATCCTTCTGTAAAATCTTC-3'
Sl-ERF.F2	F 5'-TTGATACCACTGCTTACCTAGTTTTTCT-3'
	R 5'-TATCTTCTATGGCTCCTTCCTCTTCT-3'
Sl-ERF.F3	F 5'-AGTAGTAAGGTGACCCGGATGAAG-3'
	R 5'-CACCGATCATCCACCACAGA-3'
Sl-ERF.F4	F 5'-GAGCTAATGGCTGATTTTTGTATATAAGTTC-3'
	R 5'-AAATGGTAGAAACAGCACGAGAAAG-3'
Sl-ERF.F5	F 5'-TGGAGCGAAAGCGAAAACTAA-3'
	R 5'-GTCTGACTCGGACTCCGATTG-3'
Sl-ERF.G1	F 5'-GAAGAAAGCGATCGATTTGAAGA-3'
	R 5'-TTTTCCCCATGGCCTCTGT-3'
Sl-ERF.G2	F 5'-CGGTGGAGATAAAAGCGAAAAC-3'
	R 5'-CCACTTCGCAGAACCCTAGATT-3'
Sl-ERF.H1	F 5'-AGATGCAGCAAGAGCATATGATG-3'

	R 5'-TTGGGTTGTATGGGAAATTAGTTCT-3'
β-LCY1	F 5'-GTCCACTTCCAGTATTACCTCAG-3'
	R 5'-TGTCCTTGCCACCATATAACC-3'
β-LCY2	F 5'-CGGGTTATATGGTAGCAAGGA-3'
	R 5'-CAGATGCCGATAACTCATTACC-3'
PSY1	F 5'-GGAAAGCAAACTAATAATGGACGG-3'
	R 5'-CCACATCATAGACCATCTGTTCC-3'
PDS	F 5'-GGTCACAAACCGATACTGCT-3'
	R 5'-AAACCAGTCTCGTACCAATCTC-3'
CYC-β	F 5'-TGTTATTGAGGAAGAGAAATGTGTGAT-3'
	R 5'-TCCCACCAATAGCCATAACATTTT-3'
ZDS	F 5'-AGTGGTTTCTGTCTAAAGGTGG-3'
	R 5'-ACCGAGCACTCATGTTATCAC-3'
PG2a	F 5'-TCAAGGGCACAAGTGCAACAAAGG-3'
	R 5'-TGCACGTAGCCTCTGATGGTTT-3'
RIN	F 5'-ATGCAGCACCATCAACACAT-3'
	R 5'-CTCCAAATTCAAAGCATCCA-3'
NOR	F 5'-AGAGAACGATGCATGGAGGTTTGT-3'
	R 5'-ACTGGCTCAGGAAATTGGCAATGG-3'
FUL1	R 5'-GTTTTGCCACAACAACTGGACTC-3'
	R 5'-CTTGCTGCTGTGAAGAACTACC-3'
FUL2	R 5'-AATGGAGAAGTAGAAGGATCATCG-3'
	R 5'-GATAACATAATATTGTCCGCTTGC-3'
CNR	F 5'-GCCAAATCAAGCAATGATGA-3'
	R 5'-TCGCAACCATACAGACCATT-3'
TAGL1	F 5'-ACTTTCTGTTCTTTGTGATGCT-3'
	R 5'-TTGGATGCTTCTTGCTGGTAG-3'
AP2a	F 5'-AACGGACCACAATCTTGAC-3'
	R 5'-CTGCTCGGAGTCTGAACC-3'

HB-1	F 5'-CAATCGGAGGAAGATGATGG-3'
	R 5'-TGTTCATGGTGCTGCTCTTC-3'
