# Supplementary figures and images for: Auxin Response Factor SlARF2 Is an Essential Component of the Regulatory Mechanism Controlling Fruit Ripening in Tomato
Source: PLoS Genet. 2015 Dec 30;11(12):e1005649. doi: 10.1371/journal.pgen.1005649 (PMC4696797; doi:10.1371/journal.pgen.1005649)

A

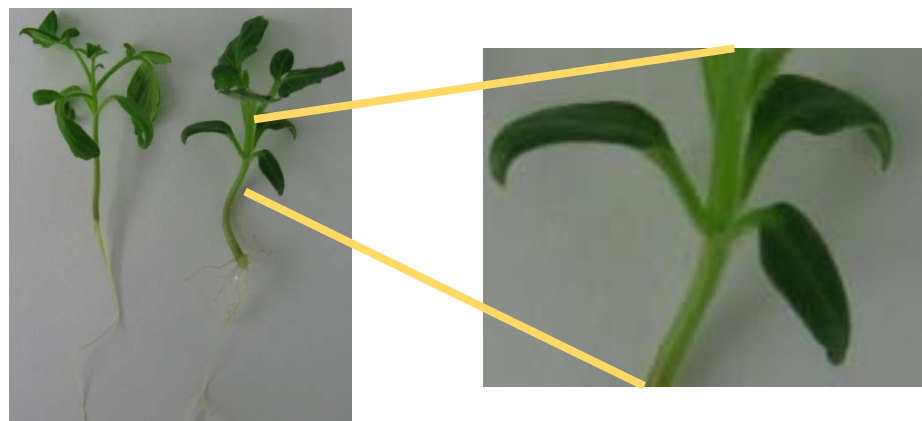

WT *SIARF2B-RNAi*  
(B1)

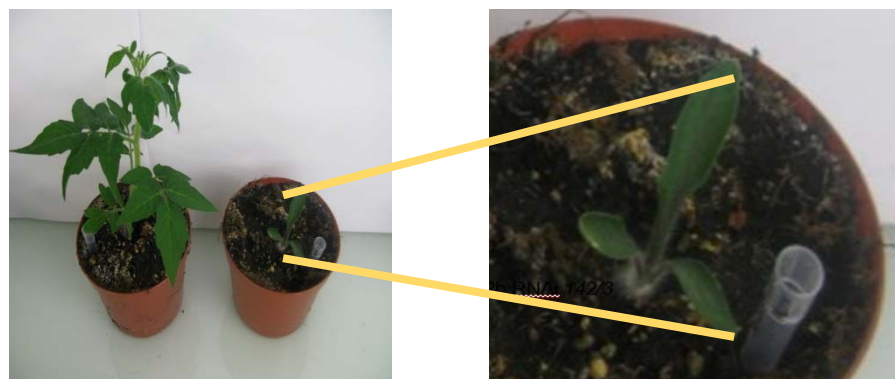

WT *SIARF2AB-RNAi*  
(AB1)

B

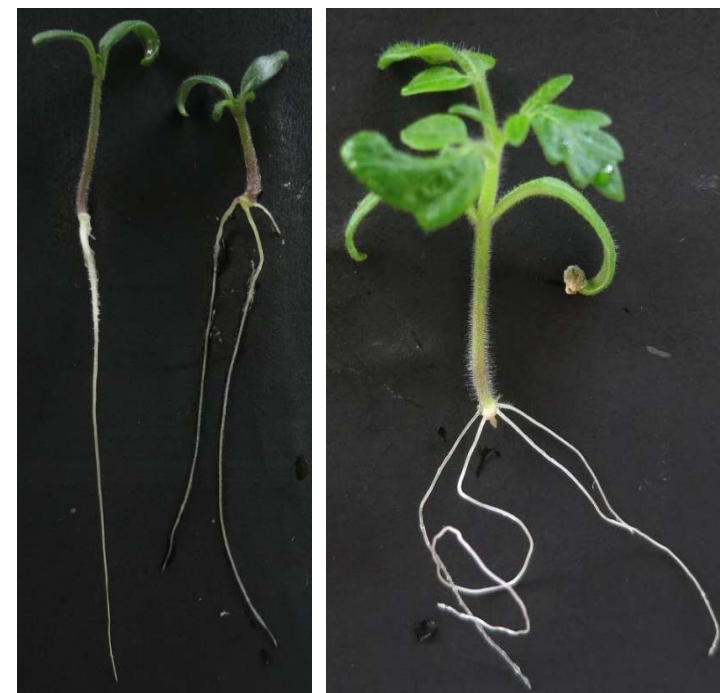

WT

*SIARF2B-RNAi*  
(B1)

Supplement: S1 Fig — (A) SlARF2A/B-RNAi lines showing the development of triple cotyledons. (B) SlARF2A/B lines showing root branching phenotypes. (PDF) [file pgen.1005649.s002.pdf]

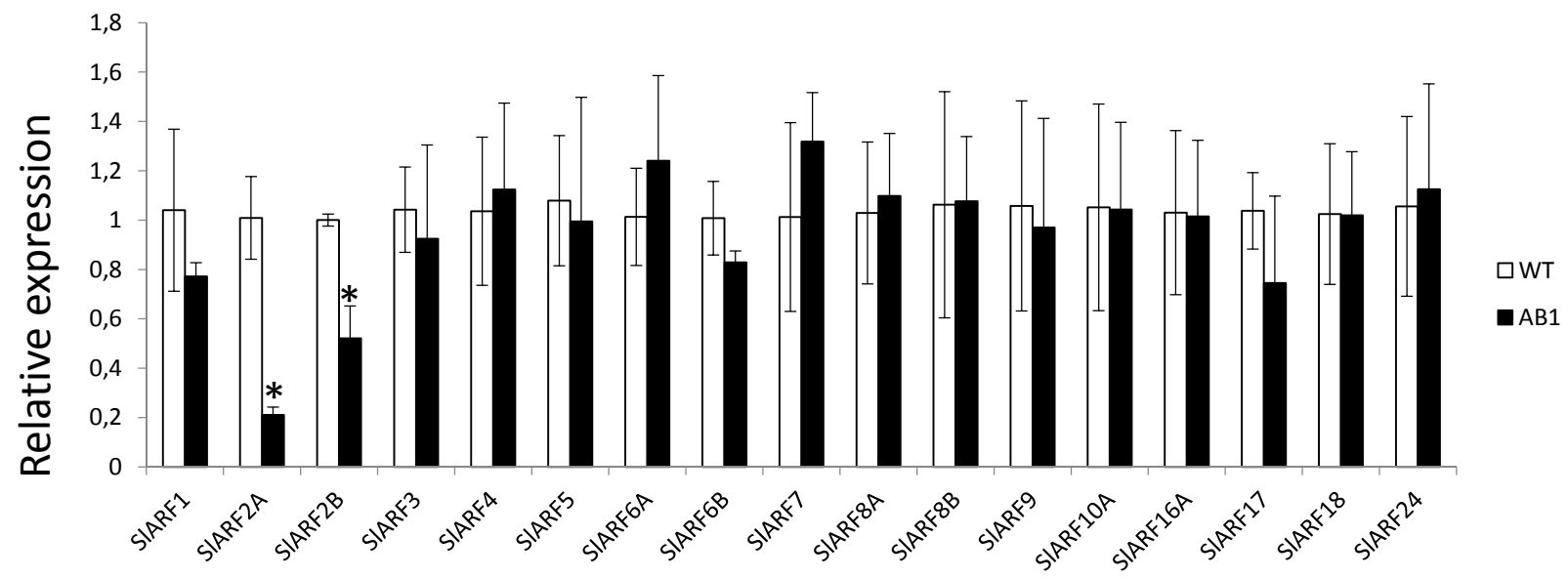

Supplement: S2 Fig — Total RNA was extracted from WT and mutant fruits at the breaker stage. The relative mRNA levels of each SlARF gene in WT were standardized to 1.0, referring to the SlActin gene as internal control. Error bar means ±SD of three biological replicates. Stars indicate statistical significance using Student’s t-test: * p-value<0.05, AB1 refers to SlARF2AB-RNAi line 311. (PDF) [file pgen.1005649.s003.pdf]
